# Setting Research Priorities to Reduce Mortality and Morbidity of Childhood Diarrhoeal Disease in the Next 15 Years

**DOI:** 10.1371/journal.pmed.1001446

**Published:** 2013-05-14

**Authors:** Kerri Wazny, Alvin Zipursky, Robert Black, Valerie Curtis, Christopher Duggan, Richard Guerrant, Myron Levine, William A. Petri, Mathuram Santosham, Rebecca Scharf, Philip M. Sherman, Evan Simpson, Mark Young, Zulfiqar A. Bhutta

**Affiliations:** 1 Programme for Global Paediatric Research, the Hospital for Sick Children, Toronto, Canada; 2 Department of International Health, Bloomberg School of Public Health, Johns Hopkins University, Baltimore, United States of America; 3 Hygiene Centre, London School of Hygiene and Tropical Medicine, London, United Kingdom; 4 Center for Nutrition, Division of GI/Nutrition, Boston Children's Hospital, Boston, United States of America; 5 Center for Global Health, Division of Infectious Diseases and International Health, University of Virginia, Charlottesville, United States of America; 6 Center for Vaccine Development, University of Maryland School of Medicine, Baltimore, United States of America; 7 Division of Infectious Diseases and International Health, University of Virginia, Charlottesville, United States of America; 8 Department of Pediatrics, University of Virginia School of Medicine, Charlottesville, United States of America; 9 Canadian Institutes of Health Research Institute of Nutrition, Metabolism, and Diabetes, Toronto, Canada; 10 PATH, Seattle, United States of America; 11 Programme Division, United Nations Children's Fund, New York, United States of America; 12 Division of Women and Child Health, Aga Khan University Hospital, Karachi, Pakistan; 13 Global Child Health, the Hospital for Sick Children, Toronto, Canada

## Abstract

Zulfi Bhutta and colleagues lay out research priorities for global child diarrheal disease over the next 15 years, which they developed using the Child Health and Nutrition Research Initiative (CHNRI) method.

*Please see later in the article for the Editors' Summary*

**Summary Points**

- This paper aims to identify research priorities, using the Child Health and Nutrition Research Initiative's (CHNRI's) method, for global childhood diarrhoeal disease over the next 15 years.
- Ten teams were established, and over 150 experts participated on one or more teams, generating and scoring 466 research questions.
- Research questions involving improving implementation, especially through behaviour change and other delivery strategies ranked highly; oral rehydration and zinc were also seen as priorities, as research questions asking to identify driving factors of caregiver demand for oral rehydration solution (ORS) and zinc and development of an ORS formulation that reduces stool output were ranked highly.
- Despite a range of discovery-related research topics, implementation research questions related to known interventions for childhood diarrhoeal diseases were ranked highly by most experts.
- In tandem with the Global Action Plan for Pneumonia and Diarrhoea, concerted efforts by a range of stakeholders in implementation research will be needed to equitably scale up already proven, effective interventions.

## Introduction

While considerable progress has been made towards the Millennium Development Goals (MDGs) and childhood diarrheal diseases have reduced from 4.6 million to 0.8 million over the last three decades, the number of diarrhoeal deaths remains unacceptably high [1]–[8]. These deaths remain concentrated in a relatively small number of countries and in poor and difficult-to-reach populations. For diarrhoeal disease in particular, coverage indicators for key preventive and curative interventions remain suboptimal, suggesting that efforts to reduce diarrhoea-related child deaths by two-thirds have stalled [4],[5],[9]–[14]. Moreover, although deaths have declined globally, the proportion of decline has been greater in high-income countries, suggesting that significant inequities between the developed and developing countries have persisted [6].

As major growth and brain development occur in the first two years of life, the impact of diarrhoeal morbidity on disability-adjusted life years (DALYs) is likely to remain substantial even as diarrhoeal mortality diminishes following current trends [15]–[17]. Nutritional deficits caused by diarrhoea can affect a child's growth, fitness, cognition, and performance at school [15],[17]. It is estimated that each diarrhoeal episode experienced by a child in the months preceding the child's second birthday increases the risk of being stunted by 5% [18]. Moreover, diarrhoeal illness in early childhood is associated with long-term adverse cognitive effects and decreased work productivity later in life [15].

Trends in coverage of many life-saving interventions have varied [9],[10]. Santosham et al. [12] reported that from 1982 to 1988 the proportion of children under age 5 receiving oral rehydration solution (ORS) grew from 5% to 60% as a result of substantial investment in diarrhoeal control programs. During this time, Brazil and Egypt made enormous progress in reducing childhood mortality due to diarrhoea [12],[19], reporting a 67% and 74% reduction, respectively. Regrettably, the median coverage of ORS has dropped since the late 1980s. Currently, the median percentage of access to ORS is 30.9% [13],[14]. The previous Countdown Report, published in 2008, combined data for ORS and ORS with continued feeding, reporting a median coverage of 38% with a range of 7%–76% [9]. Thus, coverage indicators for diarrhoea treatment have not progressed over the past four years.

## Mismatch of Burden and Investments in Programs and Research

Investment into diarrhoeal disease control has been disproportionately low in comparison to other diseases, and coverage has stalled [4],[12],[20]. Only about 2 cents for every 10 US dollars in health research annually is allocated to pneumonia and diarrhoea [21]. While diarrhoeal disease control programs receive US $10 per DALY globally, diabetes and cardiovascular disease programmes receive US $102.07 per DALY and US $63.45 per DALY, respectively [4],[20]. A recognized limitation of research investments in the area relate to lack of consensus on priorities.

The 2009 Global Forum Report on financial flows in health research highlights the importance of researchers and policy makers ensuring economic and social returns on research. It emphasizes transparency in investments and attention to health inequities [22]. The Child Health and Nutrition Research Initiative (CHNRI) developed a method to systematically and transparently identify research gaps and resource priorities [4],[7],[23]–[27]. Two previous exercises have employed the CHNRI method to identify research priorities to reduce mortality from childhood diarrhoea by 2015, but these previous CHNRI exercises had several limitations [4],[23]. Firstly, the main focus was on mortality and disease burden within the time frame of the MDGs, i.e. 2015, which influenced the choice of interventions and research options. The exercises were also limited to 10 [23] and 13 [4] participants, and 46 and 154 scored research priorities, respectively. Given the need to focus on achieving major reduction in diarrheal deaths and morbidity over a longer time frame to make it consistent with the Global Action Plan for Pneumonia and Diarrhoea (GAPPD) [28], we undertook a fresh exercise building and expanding the previous two exercises in terms of the timeframe for the research options, number of research options generated, and number of participants. We employed the CHNRI method to identify research gaps and resource priorities to reduce *morbidity* and mortality caused by childhood diarrhoeal disease over the next 15 years.

## Methods

The CHNRI methodology was created to assist those who develop research policy and/or invest in health research by identifying research gaps and resource priorities systematically and transparently in a specified context [26],[27],[29]. The aim is to help policy makers understand the potential risks and benefits of a range of research options. This methodology has been used previously to identify research gaps and resource priorities in areas such as birth asphyxia and childhood pneumonia [24],[25].

The CHNRI method has four stages: (i) the context of the problem and the criteria for priority setting are defined; (ii) technical experts generate and rank research questions; (iii) stakeholders give input regarding the weighting of the CHNRI criteria; and, (iv) research scores for the research questions are calculated and agreement between experts is analysed [25]. Detailed information on the CHNRI methodology has been provided in previous publications [24],[25]. We supplemented the CHNRI method by hosting an international workshop on the identified research priorities, which is reported elsewhere [30].

### (i) Context of the Problem Is Defined

In consideration of the substantial mortality rates of diarrhoea and its effect on morbidity as described above, we used the CHNRI method to identify research gaps and address resource priorities to questions related to both mortality and morbidity caused by childhood diarrhoeal disease. Our exercise specifically targeted a time trajectory over the next 15 years to be broadly consistent with the emerging targets for eliminating diarrhoea and pneumonia deaths by 2035 [31].

### (ii) Technical Experts Provide Input

**Figure 1:**
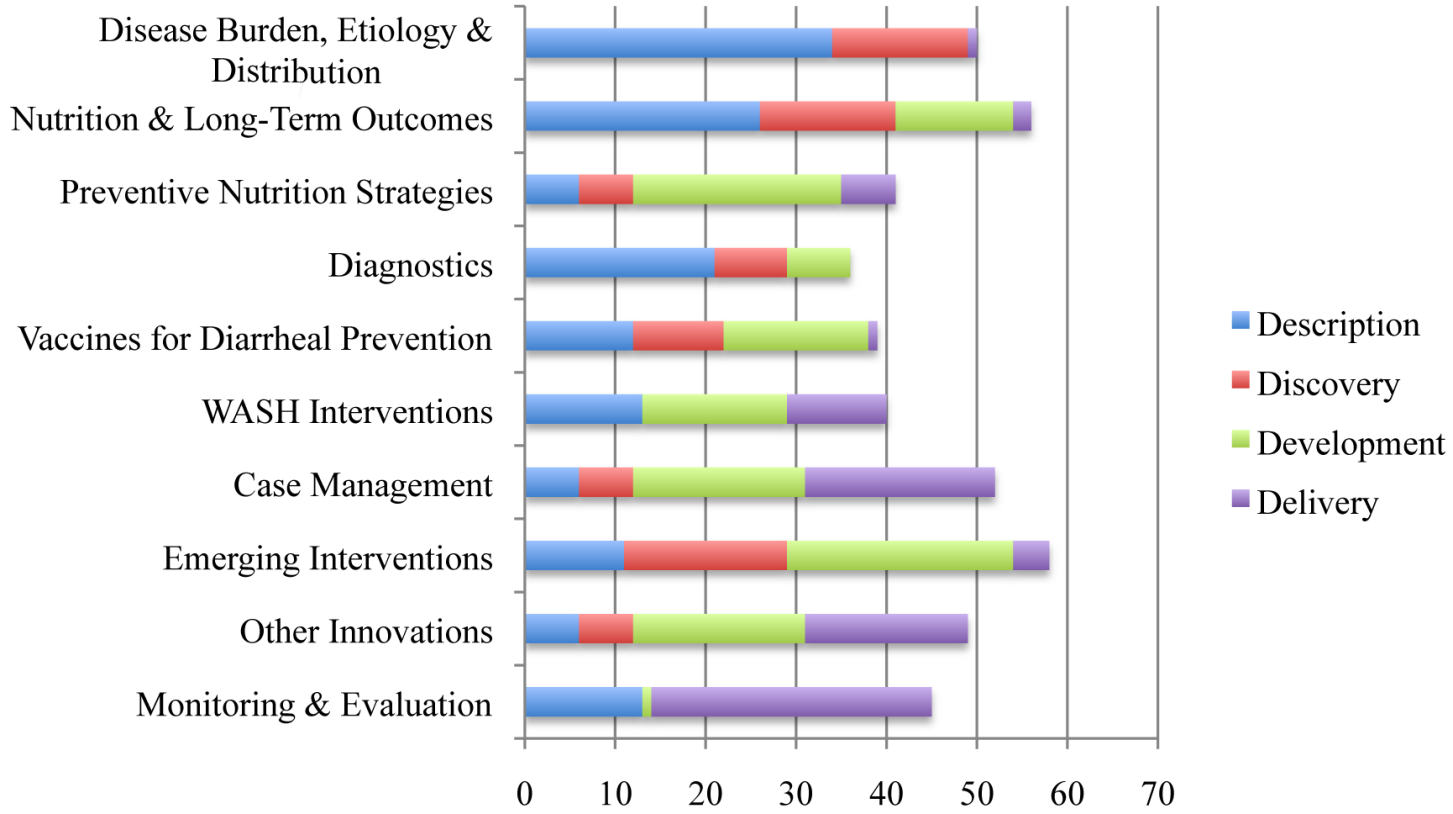
Ten “teams” of research focus and distribution of research questions into Description, Discovery, Development, and Delivery areas.

Ten areas of focus, defined by the results of the previous CHNRI exercises [4],[23], were identified and experts in each area were invited to be team leaders (Figure 1). Researchers identified team leaders by their scientific and subject expertise, contributions, and willingness to lead respective work groups. Each team leader was instructed to assemble a diverse virtual team of approximately 20 global experts representing different genders, age groups, and geographical locations. We also aimed to have representation of high-, middle- and low-income countries. Global experts were not permitted to participate in more than two teams. Information on the composition of each of the ten teams, including team leaders, global experts, countries represented, and institutional affiliations, is available in Table S16 of Supporting Information S1.

The global experts were asked to generate distinct, answerable research questions to be priorities over the next 15 years in their corresponding teams, covering the broad research domains of:

- Description (epidemiology)
- Discovery (new interventions)
- Development (improving existing interventions)
- Delivery (health policy systems, including cost-effectiveness)

These four domains (also termed D^4^), intended to be universally applicable to all health research, were proposed by the Council on Health Research and Development and modified by Rudan et al. [29]. Sample “seed questions” were provided to each team; these questions were taken from a previous CHNRI exercise to identify research priorities in childhood diarrhoea mortality [4].

Team leaders collected research questions submitted by their experts, eliminated redundancies, and chose the top questions to be scored. Research questions were scored using five criteria: (a) answerability; (b) likelihood of effectiveness; (c) likelihood of deliverability; (d) disease burden reduction; and, (e) effect on equity. A detailed description of the criteria can be found in Table S17 of Supporting Information S1. Experts were asked to score the questions, giving a 1 for yes, 0 for no, and 0.5 if they were undecided. Experts were asked to leave cells blank if they did not feel knowledgeable enough to answer the question. Blank cells did not affect the questions' scores. The Monitoring and Evaluation team recognized at an early stage that the traditional CHNRI criteria were not applicable to the research priorities their team generated; thus, this team revised some of the criteria to be more applicable to their research priorities (Table S18 of Supporting Information S1).

### (iii) External Stakeholders Agree on Weighting

Because we needed to identify a balanced portfolio of research and we lacked an empiric basis to weigh various aspects objectively, we chose to weigh all CHNRI criteria equally. This decision differs from previous CHNRI exercises where such an approach was not used [24],[25]. It was anticipated, however, that the final ranking and hierarchy of questions would reflect the priority listing of questions irrespective of individual ranking by the groups themselves.

### (iv) Research Scores Calculated for Each Team

The research priority score (RPS) and average expert agreement (AEA) were used to generate a ranking of research priorities. The RPS was computed as a mean of the scores given, by all global experts and across all criteria scored, to a particular question. The AEA score was calculated as a proportion of experts who gave the most common answer for a particular research question.

We have used an AEA to display agreement rather than a Kappa statistic, as the large number of scorers and few possible scoring options make it impossible to rule out chance, even with complete agreement among experts.

The AEA was computed using the following formula:
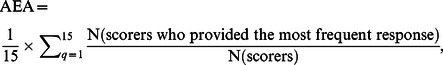
where *q* is a question that experts are being asked to evaluate competing research investment options, ranging from 1 to 15.

## Results

RPSs from the 466 questions (Table S15 of Supporting Information S1) ranged from 95.63 to 36.95 with a median of 68.50. The AEA scores ranged from 0.94 to 0.40, with a median of 0.56. The top ten questions from each team are shown in Tables S1–S10 of Supporting Information S1. The distribution of research questions into the D^4^ categories can be found in Figure 1. The top 20 research questions in the four categories are located in Tables S11–S14 of Supporting Information S1. The top 20 research questions overall are displayed in Table 1.

**Table 1:** Top 20 research questions overall.

Rank	Research Question	RPS	AEA	Team	Category
1	Identify and test alternative delivery strategies designed to ensure that ORS and zinc are reaching hard to reach populations and being used by the poorest of the poor (for example, home distribution of ORS and zinc).	95.63	0.86	Monitoring and Evaluation	Delivery
2	What are the barriers against the appropriate use of ORT?	92.14	0.74	Emerging Interventions	Description
3	What factors drive care seeking behaviour during childhood diarrhoeal disease? How can we position ORS and zinc to best respond to these factors?	91.24	0.79	Monitoring and Evaluation	Delivery
4	What factors have led to the decline in ORS use rates in countries where rates were high and now are low?	90.89	0.80	Monitoring and Evaluation	Description
5	What factors most effectively drive caregiver demand for ORS and zinc?	90.18	0.76	Monitoring and Evaluation	Delivery
6	What are the attributes of successful and sustainable childhood diarrhoea programs? E.g., what have been the design and strategies used in programs and interventions where the burden of diarrhoeal diseases has been drastically reduced?	89.16	0.68	Monitoring and Evaluation	Description
7	What is the added impact of integrated community case management on early and equitable administration of appropriate treatment for acute diarrhoea?	88.92	0.66	Monitoring and Evaluation	Delivery
8	Determine how the perception of diarrhoea as an illness affects:A. Key household practices like hand washing;B. Willingness to pay for point of use water disinfection products;C. Care seeking; and,D. Compliance to ORS and zinc treatment	88.69	0.76	Monitoring and Evaluation	Delivery
9	How do we improve the availability and uptake of interventions for diarrhoea that have consistently been shown to be effective (e.g. the 2009 WHO 7-point plan)?	88.58	0.70	Nutrition and Long-Term Outcomes	Development
10	To what extent does the roll-out of rotavirus vaccination reduce the burden of acute dehydration as well as diarrhoea?	86.46	0.73	Disease Burden, Aetiology, and Distribution	Description
11	Determine how best to move caregivers from knowledge of ORS and/or zinc treatment to actual trial and eventual adoption as routine practice. Identify the stages of behaviour change in order to tailor messages accordingly.*A.* Do we need to move from general and generic to more specific targeted messaging? When and what would this include?*B.* To move a caregiver from awareness to trial of ORS and zinc, what will be the relative impact of mass media vs. group vs. one-on-one communication strategies?*C.* Does this vary by whether rural or urban population?	86.32	0.67	Monitoring and Evaluation	Delivery
12	What are the individual risk effects of malnutrition, poor sanitation, low level of education, and reduced levels of vitamins and micronutrients in acquiring diarrhoea in children living in the developing world?	85.93	0.63	Emerging Interventions	Description
13	Can a mixture of zinc and ORS be developed that successfully reduces duration and stool output?	85.56	0.65	Other Innovations	Development
14	What contextual or cultural factors positively or negatively influence ORS and zinc utilization or compliance?	85.50	0.71	Monitoring and Evaluation	Delivery
15	What are the developmental stages/ages at which children are most at risk of long-term cognitive impacts from diarrhoea? Is there a critical window for early childhood diarrhoea that can affect future physical and mental development (0–6 m, 6 m–2 y, or 3–5 y)? (If it is greatest in the 6 m–1 y, one might place more emphasis on breast feeding and weaning practices).	84.62	0.79	Nutrition and Long-Term Outcomes	Description
16	Evaluate if early initiation and exclusive breast feeding is associated with reduced burden of diarrhoea and improved growth.	84.33	0.65	Preventive Nutrition Strategies	Description
17	Determine the best indicators for measuring the effectiveness of communication messages for childhood diarrhoea and the effectiveness of different communication channels in terms of (a) awareness of, (b) readiness to try, and (c) actual use of ORS and/or zinc.	84.18	0.69	Monitoring and Evaluation	Delivery
18	Does the community-led total sanitation approach lead to decreased diarrhoea risk?	83.96	0.65	Other Innovations	Delivery
19	Does access to, and benefits received from, nutritional supplementation programmes reduce global burden of diarrhoeal disease?	83.77	0.69	Nutrition and Long-Term Outcomes	Description
20	What are the risk factors for diarrhoea mortality?	83.41	0.69	Disease Burden, Aetiology, and Distribution	Description

### ORS/ORT and Zinc

Across the ten teams, many research questions addressed various aspects of ORS or oral rehydration therapy (ORT), many of which were also connected to zinc. The top five research questions overall were related to ORS or ORT, and had RPSs over 90.00. Of the top 50 research questions overall, 20 concerned some aspect of ORS or ORT, many of which addressed ORS/ORT and zinc concurrently. Of these 20, 13 related to some feature of delivery of ORS and ORT, such as barriers to use, driving care-seeking behaviour, and characteristics of mothers that are associated with high use of ORS/ORT. Three of the top 50 research questions concerned improving the formulation of ORS and two of the top 50 research questions concerned monitoring ORS use.

### Delivery Strategies

Questions relating to delivery strategies were found in three of four D^4^ categories. Research questions regarding descriptions of barriers to appropriate ORS use and attributes of successful programs ranked highly in the Description category. Questions regarding how to improve the uptake of the UNICEF/WHO's seven-point plan and the effect of low-cost, sustainable education packages were ranked highly in the Development category. Twenty-six of the top 50 research questions overall were either in the Delivery category, related to further developing a delivery strategy, or were of a descriptive nature that would inform a delivery strategy.

### Behaviour Change

There were numerous highly ranked questions regarding behaviour changes in mothers and other caregivers. Many research questions focused on driving care-seeking behaviour and moving caregivers from awareness to action in general and for specific interventions, including zinc, ORS, and hand washing with soap. Other behaviour change–related research priorities focused on interventions to support mothers, to encourage responsive care/parenting, or on the effect of women's peer groups and counselling on childhood diarrhoeal outcomes. Of the top 50 research questions overall, 23 involved understanding how to change the behaviour of caregivers.

### Observations on Specific Teams and D^4^ Categories

The Disease Burden, Aetiology and Distribution team prioritized developing a clear understanding of the prevalence and distribution and risk factors of diarrhoea globally. Understanding long-term child development outcomes was a priority in the Nutrition and Long-Term Outcomes team, which emphasized a need for education for diarrhoea prevention and in promoting child development.

The Preventive Nutrition Strategies team highlighted the importance of community involvement and education regarding the relationship between ideal nutrition strategies and prevention of diarrhoea. The Emerging Interventions team also prioritised research questions regarding the importance of nutritional factors in diarrhoeal disease.

The Diagnostics team prioritised research questions involving availability of technology both in diagnostic labs and in the field for accurate diagnosis of the causative agents in diarrhoea. The Vaccines for Diarrhoea Prevention team prioritized understanding barriers to effectiveness of oral vaccines in developing countries so as to develop oral vaccines with improved efficacy, and development and implementation of vaccines.

Both the Case Management team and the Monitoring and Evaluation team emphasized research priorities around ORS and zinc use, including determinants of use, factors that drive care-seeking behaviour, delivery strategies, and social marketing. The Monitoring and Evaluation team also prioritized finding factors that have led to decline in ORS use as well as defining attributes of successful and sustainable childhood diarrhoea programs.

In addition to placing an emphasis on ORS and zinc, the Other Innovations team's highly ranked questions included research questions involving feeding practices during diarrhoea and research questions regarding hand washing and sanitation. The WASH Interventions team also prioritized hand washing, highlighting the importance of better understanding the relative contribution of different transmission routes to disease burden. The team identified a need to study the effectiveness of programs to improve sanitation, water supply and hygiene behaviour in the home and in schools, and to better understand the transmission routes of diarrhoea pathogens through the environment.

Research questions in the category of Discovery tended to rank lower than those in Description, Development, and Delivery. The highest-ranked research question in the area of Discovery was twenty-third overall; the second highest Discovery question was sixty-fifth overall. Furthermore, research questions in the Diagnostics and the Vaccines for Diarrhoea Prevention areas ranked much lower relative to the research questions generated by other teams.

## Discussion

This is the largest exercise to date using the CHNRI methods and a range of subject experts building on previous exercises to develop priorities for diarrhoeal disease research. The latter utilized a more limited set of experts and specifically focused on diarrhoea mortality and morbidity in the relatively short time frame of the MDGs [4],[23]. The results of this multidisciplinary exercise emphasize strengthening the use of ORS and ORT, through new formulations, better delivery systems, and an improved understanding of the barriers to appropriate use. Research questions regarding various aspects of delivery strategies, in general, ranked highly, as did research questions regarding behaviour change. Research questions concerning new interventions ranked relatively low, which mirrors the results of the exercises conducted by Fontaine et al. and Kosek et al. [4],[23]. Fontaine and colleagues propose that the relatively low ranking of discovery-related questions was likely due to the short time frame that participants were instructed to consider [4]. However, since the time frame considered in the current exercise is one-third greater than that considered in the 2009 exercise, an alternative explanation for the low ranking of Discovery questions may be the certainty of results. As Discovery questions focused on developing entirely new interventions, research questions in Description, Development, and Delivery may have more predictable results than the Discovery research questions.

The current CHNRI exercise expanded the time lines of the previous exercise to set research priorities in childhood diarrhoeal disease over the next 15 years [4]. Fontaine et al. [4] argue that lack of implementation and coverage of cost-effective interventions, particularly in low-income settings, is a central reason that progress in preventing childhood diarrhoeal morbidity and mortality has stalled. The plentiful amount of high-priority research questions focusing on delivery of interventions supports this contention.

This CHNRI exercise differed from previous exercises by creating ten separate teams, representing different areas within the topic of diarrhoeal disease, that generated and ranked their own sets of research questions. This approach ensured a wide variety of research questions within each discipline, representation from each discipline, and experts specific to each discipline generating and ranking research questions. However, there were also disadvantages to this approach. As investments in a specific intervention can relate to multiple disciplines, there were many duplicate questions across the ten teams. Furthermore, the approach to creating research questions differed across teams; for example, research questions from the Vaccines team were much more detailed than research questions from other teams. The different styles of composing research questions could affect the scores given to those questions. Moreover, the use of multiple coordinators, some of whom had more familiarity with the CHNRI method than others, made standardization between teams challenging. Although each team was given the same format to record scores, research questions from some teams seemed to score lower as a whole than research questions from others. The research questions from the Monitoring and Evaluation team, for which custom CHNRI criteria were created (as described above), scored notably higher than research questions from other teams and 12 of the top 20 Delivery questions came from this team. Research questions from the Vaccines, Diagnostics, WASH Interventions, and Preventive Nutrition Strategies had relatively lower scores. This difference could be attributable to the research questions themselves being less important and impactful than research questions from other teams, or it could be a result of the experts being more critical scorers.

Although we strove to achieve global representation through our “global experts,” we were unable to achieve equal representation from all regions across all areas of D^4^. This could bias the types of research questions generated. In addition, questions more relevant regions that were more heavily represented through expert participation could have received higher scores as a result of this potential bias.

## Conclusions

While there is an unprecedented amount of investment in health research [10],[32], vast inequities exist in the conditions being funded. MDG 4 seeks to reduce global childhood mortality by two-thirds by 2015. Despite proven cost-effective interventions, diarrhoeal disease remains the second most important cause of death in children under five [6].

This exercise represents an important effort to assist policy-makers in identifying research gaps and resource priorities in childhood global diarrhoeal disease. Results of this CHNRI exercise emphasize a need for research to improve delivery and implementation of existing interventions.

The research priorities of this CHNRI exercise and the previous CHNRI exercise addressing childhood pneumonia [25] will be incorporated into the integrated GAPPD and will be shared with funders, donors, and science bodies over the next 24 months. The GAPPD will include contributions from key stakeholders, such as WHO, UNICEF, and USAID. Relevant stakeholders and UN agencies in six pilot countries in South Asia and East Africa have agreed to implement the GAPPD. Further development and implementation of GAPPD is planned for South Asia and East Africa.

Reducing the diarrheal morbidity and mortality in low- and middle-income countries to the levels of that in high-income countries is within our grasp. Combined efforts from all stakeholders, including donors and bilateral agencies, as well as country-level commitments and strong political will, are necessary to achieve this goal through effective implementation of currently available interventions.

## Supporting Information

Supporting Information S1 **Tables containing information about research questions and teams.** Table of contents: *Top 10 Research Questions in Each Team*. Table S1. Top 10 Research Questions in Disease Burden, Aetiology & Distribution. Table S2. Top 10 Research Questions in Nutrition & Long-Term Outcomes. Table S3. Top 10 Research Questions in Preventive Nutrition Strategies. Table S4. Top 10 Research Questions in Diagnostics. Table S5. Top 10 Research Questions in Vaccines for Diarrhoeal Prevention. Table S6. Top 10 Research Questions in WASH Interventions. Table S7. Top 10 Research Questions in Case Management. Table S8. Top 10 Research Questions in Emerging Interventions. Table S9. Top 10 Research Questions in Other Innovations. Table S10. Top 10 Research Questions in Monitoring & Evaluation. *Top Twenty Research Questions By D^4^ Category*. Table S11. Top 20 Research Questions in Description. Table S12. Top 20 Research Questions in Discovery. Table S13. Top 20 Research Questions in Development. Table S14. Top 20 Research Questions in Delivery. Table S15. All Research Questions. Table S16. Team Composition, Including Team Leaders, Participants, Countries Represented, and Institutional Affiliations. Table S17. Description of Standard CHNRI Criteria. Table S18. Description of CHNRI Criteria for Monitoring and Evaluation team.
(DOCX)

## Abbreviations

AEA: average expert agreement
CHNRI: Child Health and Nutrition Research Initiative
D^4^: Description, Discovery, Development, and Delivery
DALY: disability-adjusted life year
GAPPD: Global Action Plan for Pneumonia and Diarrhoea
MDG: Millennium Development Goal
ORS: oral rehydration solution
ORT: oral rehydration therapy
RPS: research priority score.
