# Supplementary material for: Setting Research Priorities to Reduce Mortality and Morbidity of Childhood Diarrhoeal Disease in the Next 15 Years
Source: PLoS Med. 2013 May 14;10(5):e1001446. doi: 10.1371/journal.pmed.1001446 (PMC3653794; doi:10.1371/journal.pmed.1001446)
Supplement: Supporting Information S1 — Tables containing information about research questions and teams. Table of contents: Top 10 Research Questions in Each Team. Table S1. Top 10 Research Questions in Disease Burden, Aetiology & Distribution. Table S2. Top 10 Research Questions in Nutrition & Long-Term Outcomes. Table S3. Top 10 Research Questions in Preventive Nutrition Strategies. Table S4. Top 10 Research Questions in Diagnostics. Table S5. Top 10 Research Questions in Vaccines for Diarrhoeal Prevention. Table S6. Top 10 Research Questions in WASH Interventions. Table S7. Top 10 Research Questions in Case Management. Table S8. Top 10 Research Questions in Emerging Interventions. Table S9. Top 10 Research Questions in Other Innovations. Table S10. Top 10 Research Questions in Monitoring & Evaluation. Top Twenty Research Questions By D4 Category. Table S11. Top 20 Research Questions in Description. Table S12. Top 20 Research Questions in Discovery. Table S13. Top 20 Research Questions in Development. Table S14. Top 20 Research Questions in Delivery. Table S15. All Research Questions. Table S16. Team Composition, Including Team Leaders, Participants, Countries Represented, and Institutional Affiliations. Table S17. Description of Standard CHNRI Criteria. Table S18. Description of CHNRI Criteria for Monitoring and Evaluation team. (DOCX) [file pmed.1001446.s001.docx]

**Supplementary Tables S1 - S10: Top 10 Research Questions in Each Team**

**Supplementary Table S1: Top 10 Research Questions in Disease Burden, Aetiology & Distribution**

| Rank | Research Question | RPS | AEA | Category |
| --- | --- | --- | --- | --- |
| 1 | To what extent does the roll-out of rotavirus vaccinations reduce the burden of acute dehydrating as well as all diarrhoea? | 86.46 | 0.73 | Description |
| 2 | What are the risk factors for diarrhoea mortality? | 83.41 | 0.69 | Description |
| 3 | What micronutrient deficiencies are risk factors for diarrhoea incidence and severity? | 79.74 | 0.61 | Description |
| 4 | What are the environmental and social/behavioural risk factors for diarrhoea? | 78.48 | 0.59 | Description |
| 5 | What is the best current estimate of child mortality from diarrhoea globally and in various regions of the world? | 77.88 | 0.63 | Description |
| 6 | By what mechanisms (at gut and immunologic level) do malnutrition and various micronutrient deficiencies increase risk of severe diarrhoea? | 74.94 | 0.91 | Discovery |
| 7 | Is it possible to use cross-sectional surveys to assess diarrhoea incidence and prevalence? | 74.50 | 0.89 | Description |
| 8 | Why is childhood diarrhoea mortality declining in many countries? | 74.46 | 0.59 | Description |
| 9 | What are the pathogens associated with acute and persistent diarrhoea in world regions? | 73.62 | 0.60 | Description |
| 10 | What is the incidence and prevalence of acute (without and with dehydration) diarrhoea and persistent diarrhoea in urban and rural populations and by SES? | 72.60 | 0.63 | Description |

**Supplementary Tables S1 - S10: Top 10 Research Questions in Each Team**

**Supplementary Table S2: Top 10 Research Questions in Nutrition & Long-Term Outcomes**

| Rank | Research Questions | RPS | AEA | Category |
| --- | --- | --- | --- | --- |
| 1 | How do we improve the availability and uptake of interventions for diarrhoea that have consistently been shown to be effective (e.g. the WHO 7-point plan)? | 88.58 | 0.70 | Development |
| 2 | What are the developmental stages/ages at which children are most at risk of long-term cognitive impacts from diarrhoea? Is there a critical window for early childhood diarrhoea that can affect future physical and mental development (0-6m, 6m-2y, or 3-5y)? (If it is greatest in the first six months to one year, one might place more emphasis on breast feeding and weaning practices). | 84.62 | 0.69 | Description |
| 3 | Does access to, and benefits received from, nutritional supplementation programmes reduce global burden of diarrhoeal disease? | 83.77 | 0.69 | Description |
| 4 | Do interventions to support mothers (e.g. reduce maternal depression, strengthen maternal coping, problem solving for child health) impact diarrhoeal disease outcomes? | 83.36 | 0.74 | Development |
| 5 | How do we improve the efficacy of live oral vaccines against gut or gut-acquired pathogens in low-and-middle income countries? | 82.97 | 0.61 | Discovery |
| 6 | Provision of low cost/sustainable health education packages through community involvement (community motivations steps) to mothers to prevent diarrhoea and assess effects on children’s cognition and school achievement. | 82.93 | 0.74 | Development |
| 7 | Do responsive care/parenting interventions impact diarrhoeal disease outcomes? | 81.93 | 0.73 | Development |
| 8 | Integration of health education services in health service facilities of government and NGOs and assess its cost-effectiveness on developmental outcomes. | 81.33 | 0.67 | Delivery |
| 9 | Which micronutrients or macronutrients are more effective in protecting/preventing children from enteric infections and diarrhoea early in life? | 80.51 | 0.73 | Development |
| 10 | What is the best way to develop locally appropriate and highly nutrition diets which are effective in diarrhoea management, given the great diversity of food available in low income countries? | 80.23 | 0.70 | Development |

**Supplementary Tables S1 - S10: Top 10 Research Questions in Each Team**

**Supplementary Table S3: Top 10 Research Questions in Preventive Nutrition Strategies**

| Rank | Research Questions | RPS | AEA | Category |
| --- | --- | --- | --- | --- |
| 1 | Evaluate if early initiation and exclusive breast feeding is associated with reduced burden of diarrhoea and improved growth. | 84.33 | 0.65 | Description |
| 2 | What factors, including mothers’ education, would influence acceptability of zinc supplementation and high/earlier use of ORS in the community? | 80.96 | 0.60 | Delivery |
| 3 | What is the effect of intermittent therapy with zinc on diarrhoea prevention when given at routine contacts? | 76.77 | 0.69 | Development |
| 4 | Assessment of key knowledge gaps in community awareness of the relationship between nutrition and the occurrence of diarrhoea and the relationship between diarrhoea and long-term development in children. | 76.29 | 0.54 | Delivery |
| 5 | What is the potential for women’s groups or peer-counselling/training of community-based cadres to improve infant/child nutrition and reduce diarrhoea through the uptake of preventive/therapeutic strategies? | 74.73 | 0.60 | Development |
| 6 | Determine whether iron and other micronutrient supplements reduce the effectiveness of zinc to prevent diarrhoeal disease (RCTs). | 74.23 | 0.66 | Development |
| 7 | Can complementary foods be adequately fortified with micronutrients that help reduce diarrhoea? | 73.72 | 0.65 | Development |
| 8 | Identify and test education or other interventions to reduce microbial contamination in weaning and complementary foods. | 72.70 | 0.66 | Development |
| 9 | Research to evaluate the impact of low birth weight (LBW) to subsequent malnutrition and susceptibility to diarrhoeal disease among infants and young children. | 72.65 | 0.60 | Description |
| 10 | What is the effect of routine use of preventive community supplementation of zinc on incidence and severity of diarrhoea in <36months with and without Vitamin A supplements? | 72.44 | 0.65 | Development |

**Supplementary Tables S1 - S10: Top 10 Research Questions in Each Team**

**Supplementary Table S4: Top 10 Research Questions in Diagnostics**

| Rank | Research Question | RPS | AEA | Category |
| --- | --- | --- | --- | --- |
| 1 | What are the fully burdened cost-benefits of different diagnostic technologies – molecular vs. culture vs. antigens detections vs. microscopy? | 78.31 | 0.59 | Discovery |
| 2 | To know which pathogen is the most important cause of diarrhoea in target ages, seasons and regions. | 76.95 | 0.70 | Description |
| 3 | Develop age-appropriate, geography-appropriate, duration-appropriate (acute/chronic), and characteristic-appropriate (bloody/non-bloody) algorithms for managements of different diarrhoea syndromes in different paediatric hosts. | 76.83 | 0.57 | Development |
| 4 | What are the major bacterial, viral and parasitic pathogens responsible for mortality/morbidity in acute and chronic diarrhoea among children worldwide? Are there global monitoring systems? | 76.69 | 0.68 | Description |
| 5 | How can we utilize data collected on childhood diarrhoeal disease to reduce rates infection and disease? Can this data be used to help target the development of specific vaccines, or will vaccines actually be applicable? On the other hand, can these data be used to target areas for improved hygiene/sanitation to reduce incidence? | 74.76 | 0.56 | Description |
| 6 | Develop and test an ordering algorithm for health worker/community workers/physicians for identifying causative agent of diarrhoea in an individual or outbreak situation (diagnostic test ordering algorithm). | 74.35 | 0.62 | Description |
| 7 | Can and should cheap and rapid diagnostic tests for common enteric pathogens be created for use in the field?   1. Will field or clinic of rapid diagnostic test for common enteric infections lead to improved accuracy of disease diagnosis (and more appropriate, targeted therapies or health measures) 2. Will more rapid and accurate diagnosis of enteric diseases (and use of targeted therapies) improve measures of disease outcome and long-term health? How to measure? 3. Will targeted and new diagnostics decrease antibiotic resistance? | 72.70 | 0.58 | Discovery |
| 8 | What is the added value of diagnostics for enterics from a public health cost standpoint: are certain pathogens more important to know about for prognostic/clinical reasons and what are those scenarios? | 71.51 | 0.58 | Description |
| 9 | To assess currently available tests for their sensitivity, specificity, speed and cost for diagnosis of diarrhoea. | 69.56 | 0.65 | Description |
| 10 | Develop molecular techniques for understanding co-infections (bacterial and parasitic and viral causative agents) | 67.37 | 0.53 | Discovery |

**Supplementary Tables S1 - S10: Top 10 Research Questions in Each Team**

**Supplementary Table S5: Top 10 Research Questions in Vaccines for Diarrhoeal Prevention**

| Rank | Research Questions | RPS | AEA | Category |
| --- | --- | --- | --- | --- |
| 1 | Although mucosal immune responses are elicited by oral vaccines, responses to such vaccines may be of relatively short duration compared to that induced by wild type disease. What are the reasons for this? What are the mediators and modifiers of long-term mucosal immunity? Would improved vaccines, regimens and/or immunization strategies result in longer duration? *V. cholerae* is a well studied, paradigmatic, non-invasive pathogen, and oral killed and live attenuated cholera vaccines, as well as subunit and conjugate vaccines exist. Could cholera be used as a mucosal model to address these questions? Such an approach may not only inform how to induce long-term immunity against mucosal pathogens as a group, but, if successful, could facilitate roll out and implementation of current or improved cholera vaccines. | 70.24 | 0.71 | Discovery |
| 2 | In randomized controlled field trials in sub-Saharan Africa and South and Southeast Asia, oral rotavirus vaccines have conferred ~50-60% efficacy. WHO SAGE has recommended their use and GAVI has committed to finance introduction of rotavirus vaccine into national EPIs. If a poor sub-Saharan African country achieves a high coverage of rotavirus vaccines, is it conceivable that the indirect protective effects, in addition to the direct protection, may result in a greater than expected impact on diminishing disease burden? Should it be a high priority to affirm (or disprove) this hypothesis since it has important public health implications? | 70.18 | 0.66 | Development |
| 3 | In view of clear reduced immunogenicity of oral enteric vaccines in children in developing countries, should significant resources be allocated to better understand the reason for such findings and for development of alternative modes of delivery (modified oral delivery and/or alternative routes) for efficient immunization with enteric vaccines in these populations? | 67.05 | 0.65 | Development |
| 4 | There are two licensed non-living cholera vaccines that require two doses to immunize and are useful for control of endemic disease. For control of epidemic cholera, particularly in unsettled and emergency situations, should resources be applied to complete development and achieve licensure of one or more single-dose oral cholera vaccines? | 66.72 | 0.67 | Development |
| 5 | Natural *Shigella* infection confers around 70-75% protection against the homologous serotype for a limited period of time (~2-3 years). This figure parallels the level of serum and antibody secreting cell (ASC) responses to natural infection. What would be a priority for investment of research resources: development of multicomponent (5-valent) vaccines which will cover the most common serotypes reaching this extent of protective efficacy in developing countries? And/or (?) discovery of common protein antigens (perhaps secreted proteins in vivo) which will cross-react with *Shigella* homologous and heterologous sera and further study their immunogenicity and potential to cross protect? | 66.63 | 0.67 | Development |
| 6 | Natural enterotoxinogenic Escherichia coli (ETEC) infection confers around 70-75% protection against the homologous strain for a limited period of time (~2-3 years). This figure parallels the level of serum and antibody secreting cell (ASC) responses to natural infection. What would be a priority for investment of research resources: development of a multicomponent (e.g. multivalent colonization factor antigen-based) vaccines which will cover the most common antigenic types reaching this extent of protective efficacy in developing countries? And/or (?) the discovery of common protein antigens (perhaps secreted proteins expressed in vivo) which will cross-react with ETEC homologous and heterologous sera and further study their immunogenicity and potential to cross protect? | 65.36 | 0.59 | Development |
| 7 | A constellation of data from animals and humans suggest that a pentavalent Shigella vaccine consisting of S. sonnei, S. flexneri 2a, 3a, and 6 and S. dysenteriae type 1 will cover the Shigella strains of greatest clinical and epidemiologic importance. This hypothesis, known as the "CVD pentavalent strategy" is based on finding suggesting that two of the three S. flexneri serotypes (2a and 3a) slated for inclusion in the pentavalent vaccine will provide protection against the remaining 14 types and subtypes through shared group antigens. Immunization and challenge data from an animal model document the proof-of-principle of this strategy. Should selected challenge studies be performed to validate this approach in humans? | 65.33 | 0.57 | Development |
| 8 | Recent studies (see below) showed that the intradermal route of delivery (in particular, the use of microneedle devices) induce stronger mucosal and cellular immune response than intramuscular, subcutaneous and transcutaneous routes when used to deliver protein antigens (influenze hemagglutinins, HIV p24 particle protein) in mice. No studies assessed the intradermal microneedle route of delivery for polysaccharides or polysaccharide-protein conjugates. Should the intradermal microneedle delivery be extensively studied for enteric antigens like Salmonella Typhi Vi, Shigella OPS-conjugates and others as a potential solution for improved immunogenicity and efficacy of these vaccines in young children in general and in developing countries in particular? Weldon et al. Clin Vaccine Immunol. 2011; Liard et al. Vaccine 20122; Koutsonanos et al. JID 20122; JK Hickling et al. Bull WHO 2011 | 64.30 | 0.64 | Development |
| 9 | Anticipating that current vaccine development efforts will yield safe, effective and affordable ETEC and Shigella vaccines that both may exert a herd protective effect, how should these vaccines be given in endemic areas? For example, should ETEC be administered according to a standard EPI 3-dose schedule? Should Shigella be given on a modified measles schedule? Are mass vaccination days or community campaigns an option for these vaccines? | 63.73 | 0.64 | Development |
| 10 | There is accumulating evidence of the negative impact of enteric diseases occurring in early childhood on the subsequent child cognitive development. Should research resources be allocated to develop vaccine cost-effectiveness calculation models which will include this parameter as part of the burden of disease? | 63.68 | 0.61 | Description |

**Supplementary Tables S1 - S10: Top 10 Research Questions in Each Team**

**Supplementary Table S6: Top 10 Research Questions in WASH Interventions**

| Rank | Research Questions | RPS | AEA | Category |
| --- | --- | --- | --- | --- |
| 1 | What are the critical times to wash hands to reduce diarrhoeal disease? | 82.12 | 0.60 | Development |
| 2 | What are the key transmission pathways and dominant pathogens of diarrhoeal disease in different settings? | 80.93 | 0.57 | Description |
| 3 | What is the best way to improve the microbial quality of the food served to children 0-72 months at home? | 80.50 | 0.51 | Development |
| 4 | What is the impact of intermittent water supply on diarrhoeal disease and how can we ensure the microbiological quality of intermittent piped supple? | 77.05 | 0.57 | Development |
| 5 | What are the triggers of hand washing behaviour change at different occasions and for different target groups, e.g. parents, adolescents? | 76.09 | 0.55 | Development |
| 6 | What effect does the provision of sanitation and water supply in schools have on community behaviours with respect to sanitation and hygiene and what are the health outcomes for children in school and for the wider community? | 75.15 | 0.54 | Development |
| 7 | Which are the most sustainable, affordable and acceptable latrine options for the poor? | 73.90 | 0.57 | Development |
| 8 | What is the most effective mix of WASH interventions in different epidemiological settings? | 73.58 | 0.50 | Development |
| 9 | How best to identify and change the determinants of HWWS behaviours so as to get effective behaviour changes? | 73.17 | 0.44 | Delivery |
| 10 | What is the impact of water collection time (time to source) on diarrhoea? | 73.06 | 0.55 | Description |

**Supplementary Tables S1 - S10: Top 10 Research Questions in Each Team**

**Supplementary Table S7: Top 10 Research Questions in Case Management**

| Rank | Research Question | RPS | AEA | Category |
| --- | --- | --- | --- | --- |
| 1 | What is the effect of promoting a strategy asking mothers to keep ORS packets and zinc at home for use in case of diarrhoea on use and coverage, when compared to the usual strategy that requires mothers to go to a CHW or a Health Facility to obtain ORS and zinc in case of diarrhoea? | 82.89 | 0.81 | Delivery |
| 2 | Conduct social marketing research to improve acceptability of zinc treatment in the public and private sectors – packaging, language, health messages | 81.40 | 0.79 | Delivery |
| 3 | Which strategies and messages are effective in convincing health care providers of the advantage of ORS and zinc compared to antibiotics or other drugs? | 81.26 | 0.73 | Delivery |
| 4 | What is the effectiveness of iCCM in increasing coverage of zinc and ORS? | 80.33 | 0.64 | Delivery |
| 5 | Assess effectiveness of delivery strategies to provide zinc and ORS? | 79.67 | 0.76 | Delivery |
| 6 | Assess the efficacy of zinc supplementation as adjunct to standard anti-Shigella treatment on the gut mucosal and systemic response. | 78.96 | 0.75 | Development |
| 7 | What is the acceptability/adherence for zinc supplementation for the management of diarrhoea in various settings (urban, rural)? | 78.83 | 0.73 | Development |
| 8 | What are the factors driving inappropriate use of antibiotics and other inappropriate drugs by health care practitioners after over 25 years of global guidelines, national policy, training and available information, and what actions can effectively modify those inappropriate practices? | 78.63 | 0.68 | Development |
| 9 | Testing of locally adapted health messages to promote zinc and to explain zinc to mothers/users. | 76.85 | 0.73 | Delivery |
| 10 | Identify the determinants of low use of ORS/zinc among children in target populations and evaluate strategies to modify those determinants in order to increase appropriate treatment. | 76.52 | 0.71 | Description |

**Supplementary Tables S1 - S10: Top 10 Research Questions in Each Team**

**Supplementary Table S8: Top 10 Research Questions in Emerging Interventions**

| Rank | Research Question | RPS | AEA | Category |
| --- | --- | --- | --- | --- |
| 1 | What are barriers against the appropriate use of ORT? | 92.14 | 0.74 | Description |
| 2 | What are the individual risk effects of malnutrition, poor sanitation, low level of education and reduced levels of vitamins and micronutrients in acquiring diarrhoea in children living in the developed world? | 85.92 | 0.63 | Description |
| 3 | What is the role of host factors in determining diarrhoea morbidity and mortality (e.g. demographic, nutritional, genetic)? | 83.04 | 0.68 | Description |
| 4 | How best to effectively reduce the gap between knowledge and use of simple and effective interventions, such as ORS (e.g. behavioural research, product improvements)? | 82.95 | 0.68 | Delivery |
| 5 | What are the costs and benefits of education measures to decrease diarrhoeal disease in the developing world? | 81.40 | 0.63 | Delivery |
| 6 | Evaluate calcium-supplemented ORS to reduce fluid secretion through enterocyte calcium receptors. | 81.23 | 0.65 | Development |
| 7 | What are the prospects for developing new rehydration solutions for use in both developed and developing countries? | 80.32 | 0.67 | Development |
| 8 | How best to test probiotics as an adjuvant to enhance vaccines success (e.g. in a setting where efficacy of rotavirus vaccine is not fully satisfactory)? | 79.72 | 0.63 | Development |
| 9 | How do nutritional imbalances affect the frequency and quantity of acute diarrhoeal disease? | 79.47 | 0.66 | Description |
| 10 | How best to implement what is now available: vaccines, oral rehydration, referral algorithm? | 79.15 | 0.63 | Delivery |

**Supplementary Tables S1 – S10: Top 10 Research Questions in Each Team**

**Supplementary Table S9: Top 10 Research Questions in Other Innovations**

| Rank | Research Question | RPS | AEA | Category |
| --- | --- | --- | --- | --- |
| 1 | Can a mixture of zinc and ORS be developed that successfully reduces duration and stool output? | 85.56 | 0.65 | Development |
| 2 | Does the community-led total sanitation approach lead to decreased episodes? | 83.96 | 0.65 | Delivery |
| 3 | What is the impact of waterless hand sanitizer use on diarrhoea risk in household and school setting, particularly in water-constrained areas? | 82.64 | 0.63 | Development |
| 4 | Are ORT corners effective in reducing hospital admissions for severe to moderate dehydration? | 81.54 | 0.66 | Delivery |
| 5 | Could an ORS formula be developed that decreases output? | 81.41 | 0.57 | Development |
| 6 | How might HSTS demonstration at ORT corners increase uptake and use of HWTS products and subsequent reduction of diarrhoeal disease incidence in mothers presenting with infant at ORT corners? | 80.54 | 0.65 | Development |
| 7 | Does continued feeding during diarrhoea reduce weight loss for diarrhoea and risk of stunting? | 80.39 | 0.70 | Delivery |
| 8 | Which BCC/IEC messages are most effective in increasing the percentage of caregivers receiving zinc and ORS for children in the treatment of diarrhoea? | 80.03 | 0.65 | Delivery |
| 9 | What are the barriers to continued feeding by caregivers during diarrhoea episodes? | 79.58 | 0.66 | Development |
| 10 | Evaluate and address the systemic barriers to distribution of zinc through non-pharmaceutical shops and community health workers. | 77.90 | 0.53 | Delivery |

**Supplementary Tables S1 - S10: Top 10 Research Questions in Each Team**

**Supplementary Table S10: Top 10 Research Questions in Monitoring & Evaluation**

| Rank | Research Question | RPS | AEA | Category |
| --- | --- | --- | --- | --- |
| 1 | Identify and test alternative delivery strategies designed to ensure that ORS and zinc are reaching hard to reach populations and being used by the poorest of the poor (for example, home distribution of ORS and zinc). | 95.63 | 0.86 | Delivery |
| 2 | What factors drive care-seeking behaviour during childhood diarrhoeal disease? | 91.24 | 0.79 | Delivery |
| 3 | What factors have led to the decline in ORS use rates in countries where rates were high and now are low? | 90.89 | 0.79 | Description |
| 4 | What factors most effectively drive caregiver demand for ORS and zinc? | 90.18 | 0.76 | Delivery |
| 5 | What are the attributes of successful and sustainable childhood diarrhoea programs? (E.g. what have been the design and strategies used in programs and interventions where the burden of diarrhoeal diseases has been drastically reduced?) | 89.16 | 0.68 | Description |
| 6 | What is the added impact of integrated community case management on early and equitable administration of appropriate treatment for acute diarrhoea? | 88.92 | 0.66 | Delivery |
| 7 | Determine how the perception of diarrhoea as an illness affects:   1. Key household practices like hand washing; 2. Willingness to pay for point of use water disinfection products; 3. Care seeking; and, 4. Compliance to ORS and zinc treatment. | 88.69 | 0.76 | Delivery |
| 8 | Determine how best to move caregivers from knowledge of ORS and/or zinc treatment to actual trial and eventual adoption as routine practice. Identify the stages of behaviour change in order to tailor messages accordingly.   1. Do we need to move from general and generic to more specific, targeted messaging? When and what would this include? 2. To move a caregiver from awareness to trial of ORS and zinc, what will be the relative impact of mass media vs. group vs. one-on-one communication strategies? 3. Does this vary by whether a rural or urban population? | 86.32 | 0.67 | Delivery |
| 9 | What contextual or cultural factors positively or negatively influence ORS and zinc utilization or compliance? | 85.50 | 0.71 | Delivery |
| 10 | Determine the best indicators for measuring the effectiveness of communication messages for childhood diarrhoea and the effectiveness of different communication channels in terms of a) awareness, b) readiness to try, and c) actual use of ORS and/or zinc | 84.14 | 0.69 | Delivery |

**Supplementary Tables S11 - S14 – Top Twenty Research Questions By ^D4^ Categories**

**Supplementary Table S11: Top 20 Research Questions in Description**

| Rank | Team | Research Question | RPS | AEA | (Rank in team) |
| --- | --- | --- | --- | --- | --- |
| 1 | Emerging Interventions | What are the barriers against the appropriate use of ORT? | 92.14 | 0.74 | 1 |
| 2 | Monitoring & Evaluation | What factors have led to the decline in ORS where rates were high and now are low? | 90.89 | 0.80 | 3 |
| 3 | Monitoring & Evaluation | What are the attributes of successful and sustainable childhood diarrhoea programs? E.g. what have been the design and strategies used in programs and interventions where the burden of diarrhoeal diseases has been drastically reduced? | 89.16 | 0.68 | 5 |
| 4 | Disease Burden, Etiology and Distribution | To what extent does the roll-out of rotavirus vaccination reduce the burden of acute dehydration as well as all diarrhoea? | 86.46 | 0.73 | 7 |
| 5 | Emerging Interventions | What are the individual risk effects of malnutrition, poor sanitation, low level of education and reduced levels of vitamins and micronutrients in acquiring diarrhoea in children living in the developing world? | 85.93 | 0.63 | 2 |
| 6 | Nutrition & Long-Term Outcomes | What are the developmental stages/ages at which children are most at risk of long term cognitive impacts from diarrhoea? Is there a critical window for early childhood development (0-6m, 6m-2y, or 3-5y)? (If it is greatest in the first 6m-1y, one might place more emphasis on breast feeding and weaning practices) | 84.62 | 0.79 | 2 |
| 7 | Preventive Nutrition Strategies | Evaluate if early initiation and exclusive breast feeding is associated with reduced burden of diarrhoea and improved growth. | 84.33 | 0.65 | 1 |
| 8 | Nutrition & Long-Term Outcomes | Does access to, and benefits received from, nutritional supplementation programmes reduce global burden of diarrhoeal disease? | 83.77 | 0.69 | 3 |
| 9 | Disease Burden, Aetiology and Distribution | What are the risk factors for diarrhoea mortality? | 83.41 | 0.69 | 2 |
| 10 | Emerging Interventions | What is the role of host factors in determining diarrhoea morbidity and mortality? (e.g. demographic, nutritional, genetic) | 83.04 | 0.68 | 3 |
| 11 | WASH Interventions | What are the key transmission pathways and dominant pathogens of diarrhoeal disease in different settings? | 80.93 | 0.57 | 2 |
| 12 | Monitoring & Evaluation | What is the sensitivity and specificity of the current home oral rehydration treatment and ORS questions in DHS and MICS and are there better questions to measure use of ORS? | 80.55 | 0.67 | 15 |
| 13 | Disease Burden, Aetiology & Distribution | What micronutrient deficiencies are risk factors for diarrhoea incidence or severity? | 79.74 | 0.61 | 3 |
| 14 | Nutrition & Long-Term Outcomes | How does childhood diarrhoeal illness correlate with adult height? What is the impact of acute, prolonged, persistent and recurrent diarrhoea on growth trajectories of children in impoverished endemic areas? | 79.70 | 0.65 | 12 |
| 15 | Emerging Interventions | How do nutritional imbalances affect the frequency and quantity of acute diarrhoeal disease? | 79.47 | 0.66 | 9 |
| 16 | Nutrition & Long-Term Outcomes | What would be the long-term costs to human capital of preventing diarrhoea mortality without preventing diarrhoea morbidity? | 79.15 | 0.51 | 14 |
| 17 | Disease Burden, Aetiology and Distribution | What are the environmental and social/behavioural risk factors for diarrhoea? | 78.48 | 0.59 | 4 |
| 18 | Nutrition & Long-Term Outcomes | To what extent does the connection between childhood diarrhoea and undernutrition contribute to lethal comorbidities such as pneumonia, malaria, TB? | 78.22 | 0.73 | 18 |
| 19 | Disease Burden, Aetiology & Distribution | What is the best current estimate of child mortality from diarrhoea globally and in various regions in the world? | 77.88 | 0.63 | 5 |
| 20 | Emerging Interventions | Why do some children with acute diarrhoea go on to develop chronic diarrhoea and what can be done to prevent this? | 77.80 | 0.66 | 13 |

**Supplementary Tables S11 - S14 – Top Twenty Research Questions By D^4^ Categories**

**Supplementary Table S12: Top 20 Research Questions in Discovery**

| Rank | Team | Research Question | RPS | AEA | Rank in Team |
| --- | --- | --- | --- | --- | --- |
| 1 | Nutrition & Long-Term Outcomes | How do we improve the efficacy of live oral vaccines against gut or gut acquired pathogens in low-and-middle-income countries? | 82.97 | 0.61 | 5 |
| 2 | Nutrition & Long-Term Outcomes | Develop a successful vaccine against Cryptosporidium, Shigella, Giardia. | 78.88 | 0.55 | 16 |
| 3 | Nutrition & Long-Term Outcomes | Research suggests that cognitive deficits associated with early childhood diarrhoea persist for at least 4 or 5 years. What measures can capture this deficit? How long does this deficit persist?^[[1]](#footnote-1)^ | 78.57 | 0.69 | 17 |
| 4 | Diagnostics | What are the fully burdened cost-benefits of different diagnostic technologies – molecular vs. culture vs. antigen detection vs. microscopy? | 78.31 | 0.59 | 1 |
| 5 | Emerging Interventions | What is the role of short-chain fatty acid delivery to the colon in enhancing sodium and water absorption, reducing fluid secretion and facilitating mucosal repair? | 77.98 | 0.61 | 11 |
| 6 | Disease Burden, Aetiology & Distribution | By what mechanisms (at gut and immunologic level) do malnutrition and various micronutrient deficiencies increase risk of severe diarrhoea? | 74.94 | 0.91 | 6 |
| 7 | Emerging Interventions | Identify and validate biomarkers of ‘gut health’ (e.g. gut barrier function, inflammatory biomarkers, etc.) to identify those children at risk of chronic enteropathy. | 74.81 | 0.58 | 22 |
| 8 | Other Innovations | What is the role of Vitamin D deficiency in diarrhoea risk? | 74.61 | 0.56 | 15 |
| 9 | Case Management | Study the effect of zinc on the gut secretory mechanisms. | 74.42 | 0.71 | 16 |
| 10 | Emerging Interventions | What is the role of co-infections in childhood diarrhoea? | 73.32 | 0.63 | 24 |
| 11 | Other Innovations | Can a water storage vehicle be developed with slow release halogen? | 72.77 | 0.53 | 16 |
| 12 | Diagnostics | Can and should cheap and rapid diagnostic tests for common enteric pathogens be created for use in the field?   1. Will field or clinic use of rapid diagnostic test for common enteric infections lead to improved accuracy of disease diagnosis (and more appropriate, targeted therapies or health measures)? 2. Will more rapid and accurate diagnosis of enteric diseases (and use of targeted therapies) improve measures of disease outcome and long-term health? How to measure? 3. Will targeting therapies and new diagnostics decrease antibiotic resistance? | 72.70 | 0.58 | 7 |
| 13 | Emerging Interventions | What is the effect of enteral glutamine on mucosal and systemic immune responses in children with diarrhoea? | 72.65 | 0.60 | 26 |
| 14 | Emerging Interventions | Assess the utility of targeting NKCC, K channels and Na-coupled transporters in diarrhoea therapy. | 72.19 | 0.58 | 27 |
| 15 | Nutrition and Long-Term Outcomes | What is the better approach to improve the intestinal microbiome in order to enrich the nutrient absorption and protect the intestinal barrier function following enteric infections? | 70.95 | 0.51 | 34 |
| 16 | Disease Burden, Aetiology & Distribution | Are the enteropathogens (particularly chronic infections for which treatment exists, i.e. helminths) that modulate the incidence and severity of other enteropathogens? | 70.59 | 0.55 | 15 |
| 17 | Vaccines | Although mucosal immune responses are elicited by oral vaccines, responses to such vaccines may be of relatively short duration compared to that induced by wild type disease. What are the reasons for this? What are the mediators and modifiers of long-term mucosal immunity? Would improved vaccines, regimens and/or immunization strategies result in longer duration? *V. cholerae* is a well studied, paradigmatic, non-invasive pathogen, and oral killed and live attenuated cholera vaccines, as well as subunit and conjugate vaccines exist. Could cholera be used as a musical model to address these questions? Such an approach may not only inform how to induce long-term immunity against mucosal pathogens as a group, but, if successful, could facilitate roll-out and implementation of current or improved cholera vaccines. | 70.24 | 0.71 | 1 |
| 18 | Diagnostics | Develop molecular techniques for understanding co-infections (bacterial and parasitic and viral causative agents). | 67.37 | 0.53 | 10 |
| 19 | Disease Burden, Aetiology & Distribution | How do age, aetiology and severity of diarrhoea affect the integrity of the gut and subsequent growth/health? | 66.82 | 0.56 | 20 |
| 20 | Emerging Interventions | Establish the role of chloride channels in rotavirus-induced diarrhoea and then develop and test inhibitors of calcium activated chloride channels. | 66.68 | 0.57 | 36 |

**Supplementary Tables S11 - S14 – Top Twenty Research Questions By D^4^ Categories**

**Supplementary Table S13: Top 20 Research Questions in Development**

| Rank | Team | Research Question | RPS | AEA | Rank in team |
| --- | --- | --- | --- | --- | --- |
| 1 | Nutrition & Long-Term Outcomes | How do we improve the availability and uptake of interventions for diarrhoea that have consistently been shown to be effective (e.g. the 2009 WHO 7-point plan)? | 88.58 | 0.70 | 1 |
| 2 | Other Innovations | Can a mixture of zinc and ORS be developed that successfully reduces duration and stool output? | 85.56 | 0.65 | 1 |
| 3 | Nutrition & Long-Term Outcomes | Do interventions to support mothers (e.g. reduce maternal depression, strengthen maternal coping, problem solving for child health) impact diarrhoeal disease outcomes? | 83.36 | 0.74 | 4 |
| 4 | Nutrition & Long-Term Outcomes | Provision of low cost/sustainable health education packages through community involvement (community motivation steps) to mothers to prevent diarrhoea and assess effects on children’s cognition and school achievement. | 82.93 | 0.74 | 6 |
| 5 | Other Innovations | What is the impact of waterless hand sanitizer use on diarrhoea risk in household and school settings, particularly in water-constrained areas? | 82.64 | 0.63 | 3 |
| 6 | WASH Interventions | What are the critical times to wash hands to reduce diarrhoeal disease? | 82.12 | 0.60 | 1 |
| 7 | Nutrition & Long-Term Outcomes | Do responsive care/parenting interventions impact diarrhoeal disease outcome? | 81.93 | 0.73 | 7 |
| 8 | Other Innovations | Could an ORS formula be developed that decreases output? | 81.41 | 0.57 | 5 |
| 9 | Emerging Interventions | Evaluate calcium-supplemented ORS to reduce fluid secretion through enterocyte calcium receptors. | 81.23 | 0.65 | 6 |
| 10 | Other Innovations | How might HWTS demonstration at ORT corners increase uptake and use of HWTS products and subsequent reduction of diarrhoeal disease incidence in mother presenting with infants at ORT corners? | 80.54 | 0.65 | 6 |
| 11 | Nutrition & Long-Term Outcomes | Which micronutrients or macronutrients are more effective in protecting/preventing children from enteric infections and diarrhoea early in life? | 80.51 | 0.73 | 9 |
| 12 | WASH Interventions | What is the best way to improve the microbial quality of the food served to children 0-72months at home? | 80.50 | 0.51 | 3 |
| 13 | Emerging Interventions | What are the prospects for developing new rehydration solutions for use in both developed and developing countries? | 80.32 | 0.67 | 7 |
| 14 | Nutrition & Long-Term Outcomes | What is the best way to develop locally appropriate and highly nutritious diets, which are effective in diarrhoea management, given the great diversity of food available in low income countries? | 80.23 | 0.70 | 10 |
| 15 | Emerging Interventions | How best to test probiotics as an adjuvant to enhance vaccine success (e.g. in a setting where efficacy of rotavirus vaccine is not fully satisfactory)? | 79.72 | 0.65 | 8 |
| 16 | Other Innovations | What are the barriers to continued feeding by caregivers during diarrhoea episodes? | 79.58 | 0.66 | 8 |
| 17 | Nutrition & Long-Term Outcomes | Awareness raising programs using media involvement (posters, banners, dramas, radio, TV, flip chart, etc.) to combat/prevent diarrhoea and the effects on childhood cognition and cost analysis. | 79.46 | 0.65 | 13 |
| 18 | Case Management | Assess the efficacy of zinc supplementation as an adjunct to standard anti-Shigella treatment on the gut mucosal and systemic response. | 78.96 | 0.75 | 6 |
| 19 | Nutrition & Long-Term Outcomes | Do responsive feeding interventions impact on diarrhoeal disease outcomes? | 78.93 | 0.62 | 15 |
| 20 | Case Management | What is the acceptability/adherence for zinc supplementation for the management of diarrhoea in various settings (urban, rural)? | 78.83 | 0.73 | 7 |

**Supplementary Tables S11 - S14 – Top Twenty Research Questions By D^4^ Categories**

**Supplementary Table S14: Top 20 Research Questions in Delivery**

| Rank | Team | Research Question | RPS | AEA | Rank in Team |
| --- | --- | --- | --- | --- | --- |
| 1 | Monitoring & Evaluation | Identify and test alternative delivery strategies designed to ensure that ORS and zinc are reaching hard to reach populations and being used by the poorest of the poor (for example, home distribution of ORS and zinc). | 95.63 | 0.86 | 1 |
| 2 | Monitoring & Evaluation | What factors drive care seeking behaviour during childhood diarrhoeal disease? How can we position, ORS and zinc to best respond to these factors? | 91.24 | 0.79 | 2 |
| 3 | Monitoring & Evaluation | What factors most effectively drive caregiver demand for ORS and zinc? | 90.18 | 0.76 | 4 |
| 4 | Monitoring & Evaluation | What is the added impact of integrated community case management on early and equitable administration of appropriate treatment for acute diarrhoea? | 88.92 | 0.66 | 6 |
| 5 | Monitoring & Evaluation | Determine how the perception of diarrhoea as an illness affects:   1. Key household practices like hand washing; 2. Willingness to pay for point of use water disinfection products; 3. Care seeking; and, 4. Compliance to ORS and zinc treatment | 88.69 | 0.76 | 7 |
| 6 | Monitoring & Evaluation | Determine how best to move caregivers from knowledge of ORS and/or zinc treatment to actual trial and eventual adoption as routine practice. Identify the stages of behaviour change in order to tailor messages accordingly.   1. Do we need to move from general and generic to more specific targeted messaging? When and what would this include? 2. To move a caregiver from awareness to trial of ORS and zinc, what will be the relative impact of mass media vs. group vs. one-on-one communication strategies? 3. Does this vary by whether a rural or urban population? | 86.32 | 0.67 | 8 |
| 7 | Monitoring & Evaluation | What contextual or cultural factors positively or negatively influence ORS and zinc utilization or compliance? | 85.50 | 0.71 | 9 |
| 8 | Monitoring & Evaluation | Determine the best indicators for measuring the effectiveness of communication messages for childhood diarrhoea and the effectiveness of different communication channels in terms of a) awareness; b) readiness to try; and, c) actual use of ORS and/or zinc. | 84.18 | 0.69 | 10 |
| 9 | Other Innovations | Does the community-led total sanitation approach lead to decreased diarrhoea risk? | 83.96 | 0.65 | 2 |
| 10 | Emerging Interventions | How best to effectively reduce the gap between knowledge and use of simple and effective interventions, such as ORS (e.g. behavioural research, product improvements)? | 82.95 | 0.68 | 4 |
| 11 | Case Management | What is the effect of promoting a strategy asking mothers to keep ORS packets and zinc at home for use in case of diarrhoea on use and coverage, when compared to the usual strategy that requires mothers to go to a CHW or a Health Facility to obtain ORS and zinc in case of diarrhoea? | 82.89 | 0.81 | 1 |
| 12 | Monitoring & Evaluation | Test indicators to determine effectiveness of Integrated Management of Childhood Illnesses (IMCI) and Integrated Community Case Management (iCCM) in reducing the burden of childhood diarrhoea. | 81.75 | 0.69 | 11 |
| 13 | Monitoring & Evaluation | Evaluate alternative public sector delivery strategies, e.g. integrated community case management (iCCM), community health volunteers or workers, advance distribution through child health days or other outreach programs. What is their impact on coverage and at what cost? Would it affect other delivery outcomes? | 81.70 | 0.64 | 12 |
| 14 | Monitoring & Evaluation | What is the effectiveness in terms of supply-demand and cost of alternative distribution networks for zinc and ORS, in particular in rural or hard to reach areas? | 81.69 | 0.69 | 13 |
| 15 | Monitoring & Evaluation | Determine the best monitoring indicators to assess the full proves of program implementation (i.e. access, availability, sales of product, compliance with full 10-14 day course, geographic coverage, equity, knowledge of providers and caregivers, etc.) | 81.61 | 0.65 | 14 |
| 16 | Other Innovations | Are ORT corners effective in reducing hospital admissions for severe to moderate dehydration? | 81.54 | 0.66 | 4 |
| 17 | Case Management | Conduct social marketing research to improve acceptability of zinc treatment in the pubic and private sectors – packaging, language, health messages | 81.40 | 0.79 | 2 |
| 18 | Emerging Interventions | What are the costs and benefits of the education measures to decrease diarrhoeal disease in the developing world? | 81.40 | 0.63 | 5 |
| 19 | Nutrition & Long-Term Outcomes | Integration of health education services in health service facilities of government and NGOs and assess its cost-effectiveness on developmental outcomes | 81.33 | 0.67 | 8 |
| 20 | Case Management | Which strategies and messages are effective in convincing health care providers of the advantage of ORS and zinc compared to antibiotics or other drugs? | 81.26 | 0.73 | 3 |

**Supplementary Table S15: All Research Questions**

| Rank | Research Question | RPS | AEA | Team | Category |
| --- | --- | --- | --- | --- | --- |
| 1 | Identify and test alternative delivery strategies designed to ensure that ORS and zinc are reaching hard to reach populations and being used by the poorest of the poor (for example, home distribution of ORS and zinc). | 95.63 | 0.86 | Monitoring & Evaluation | Delivery |
| 2 | What are the barriers against the appropriate use of ORT? | 92.14 | 0.74 | Emerging Interventions | Description |
| 3 | What factors drive care seeking behaviour during childhood diarrhoeal disease? How can we position ORS and zinc to best respond to these factors? | 91.24 | 0.79 | Monitoring & Evaluation | Delivery |
| 4 | What factors have led to the decline in ORS use rates in countries where rates were high and now are low? | 90.89 | 0.80 | Monitoring & Evaluation | Description |
| 5 | What factors most effectively drive caregiver demand for ORS and zinc? | 90.18 | 0.76 | Monitoring & Evaluation | Delivery |
| 6 | What are the attributes of successful and sustainable childhood diarrhoea programs? E.g. what have been the design and strategies used in programs and interventions where the burden of diarrhoeal diseases has been drastically reduced? | 89.16 | 0.68 | Monitoring & Evaluation | Description |
| 7 | What is the added impact of integrated community case management on early and equitable administration of appropriate treatment for acute diarrhoea? | 88.92 | 0.66 | Monitoring & Evaluation | Delivery |
| 8 | Determine how the perception of diarrhoea as an illness affects:   1. Key household practices like hand washing; 2. Willingness to pay for point of use water disinfection products; 3. Care seeking; and, 4. Compliance to ORS and zinc treatment | 88.69 | 0.76 | Monitoring & Evaluation | Delivery |
| 9 | How do we improve the availability and uptake of interventions for diarrhoea that have consistently been shown to be effective (e.g. the 2009 WHO 7-point plan)? | 88.58 | 0.70 | Nutrition & Long-Term Outcomes | Development |
| 10 | To what extent does the roll-out of rotavirus vaccination reduce the burden of acute dehydration as well as diarrhoea? | 86.46 | 0.73 | Disease Burden, Aetiology & Distribution | Description |
| 11 | Determine how best to move caregivers from knowledge of ORS and/or zinc treatment to actual trial and eventual adoption as routine practice. Identify the stages of behaviour change in order to tailor messages accordingly.   1. Do we need to move from general and generic to more specific targeted messaging? When and what would this include? 2. To move a caregiver from awareness to trial of ORS and zinc, what will be the relative impact of mass media vs. group vs. one-on-one communication strategies? 3. Does this vary by whether rural or urban population? | 86.32 | 0.67 | Monitoring & Evaluation | Delivery |
| 12 | What are the individual risk effects of malnutrition, poor sanitation, low level of education and reduced levels of vitamins and micronutrients in acquiring diarrhoea in children living in the developing world? | 85.93 | 0.63 | Emerging Interventions | Description |
| 13 | Can a mixture of zinc and ORS be developed that successfully reduces duration and stool output? | 85.56 | 0.65 | Other Innovations | Development |
| 14 | What contextual or cultural factors positively or negatively influence ORS and zinc utilization or compliance? | 85.50 | 0.71 | Monitoring & Evaluation | Delivery |
| 15 | What are the developmental stages/ages at which children are most at risk of long-term cognitive impacts from diarrhoea? Is there a critical window for early childhood diarrhoea that can affect future physical and mental development (0-6m, 6m-2y, or 3-5y)? (If it is greatest in the first six months to one year, one might place more emphasis on breast feeding and weaning practices). | 84.62 | 0.79 | Nutrition & Long-Term Outcomes | Description |
| 16 | Evaluate if early initiation and exclusive breast feeding is associated with reduced burden of diarrhoea and improved growth. | 84.33 | 0.65 | Preventive Nutrition Strategies | Description |
| 17 | Determine the best indicators for measuring the effectiveness of communication messages for childhood diarrhoea and the effectiveness of different communication channels in terms of a) awareness, b) readiness to try, and c) actual use of ORS and/or zinc. | 84.18 | 0.69 | Monitoring & Evaluation | Delivery |
| 18 | Does the community-led total sanitation approach lead to decreased diarrhoea risk? | 83.96 | 0.65 | Other Innovations | Delivery |
| 19 | Does access to, and benefits received from, nutritional supplementation programmes reduce global burden of diarrhoeal disease? | 83.77 | 0.69 | Nutrition & Long-Term Outcomes | Description |
| 20 | What are the risk factors for diarrhoea mortality? | 83.41 | 0.69 | Disease Burden, Aetiology & Distribution | Description |
| 21 | Do interventions to support mothers (e.g. reduce maternal depression, strengthen maternal coping, problem solving for child health) impact diarrhoeal disease outcomes? | 83.36 | 0.74 | Nutrition & Long-Term Outcomes | Development |
| 22 | What is the role of host factors in determining diarrhoea morbidity and mortality? (e.g. demographic, nutritional, genetic) | 83.04 | 0.68 | Emerging Interventions | Description |
| 23 | How do we improve the efficacy of live oral vaccines against gut or gut-acquired pathogens in low-and-middle-income countries? | 82.97 | 0.61 | Nutrition & Long-Term Outcomes | Discovery |
| 24 | How best to effectively reduce the gap between knowledge and use of simple and effective interventions, such as ORS (e.g. behavioural research, product improvements)? | 82.95 | 0.68 | Emerging Interventions | Delivery |
| 25 | Provision of low cost/sustainable health education packages through community involvement (community motivation steps) to mothers to prevent diarrhoea and assess effects on children’s cognition and school achievement. | 82.93 | 0.74 | Nutrition & Long-Term Outcomes | Development |
| 26 | What is the effect of promoting a strategy asking mothers to keep ORS packets and zinc at home for use in case of diarrhoea on use and coverage, when compared to the usual strategy that requires mothers to go to a CHW or a Health Facility to obtain ORS and zinc in case of diarrhoea? | 82.89 | 0.81 | Case Management | Delivery |
| 27 | What is the impact of waterless hand sanitizer use on diarrhoea risk in household and school settings, particularly in water-constrained areas? | 82.64 | 0.63 | Other Innovations | Development |
| 28 | What are the critical times to wash hands to reduce diarrhoeal disease? | 82.12 | 0.60 | WASH Interventions | Development |
| 29 | Do responsive care/parenting interventions impact diarrhoeal disease outcomes? | 81.93 | 0.73 | Nutrition & Long-Term Outcomes | Development |
| 30 | Test indicators to determine effectiveness of Integrated Management of Childhood Illnesses (IMCI) and Integrated Community Case Management (iCCM) in reducing the burden of childhood diarrhoea. | 81.75 | 0.69 | Monitoring & Evaluation | Delivery |
| 31 | Evaluate alternative public sector delivery strategies, e.g. Integrated Community Case Management (iCCM), community health volunteers or workers, advance distribution through child health days or other outreach programs. What is their impact on coverage and at what cost? Would it affect other delivery outcomes? | 81.70 | 0.64 | Monitoring & Evaluation | Delivery |
| 32 | What is the effectiveness in terms of supply-demand and cost of alternative distribution networks for zinc and ORS, in particular in rural or hard to reach areas? | 81.69 | 0.69 | Monitoring & Evaluation | Delivery |
| 33 | Determine the best monitoring indicators to assess the full proves of program implementation (i.e. access availability, sales of product, compliance with full 10-14 day course, geographic coverage, equity, knowledge of providers and caregivers, etc.) | 81.61 | 0.65 | Monitoring & Evaluation | Delivery |
| 34 | Are ORT corners effective in reducing hospital admissions for severe to moderate dehydration? | 81.54 | 0.66 | Other Innovations | Delivery |
| 35 | Could an ORS formula be developed that decreases output? | 81.41 | 0.57 | Other Innovations | Development |
| 36 | Conduct social marketing research to improve acceptability of zinc treatment in the public and private sectors – packaging, language, health messages | 81.40 | 0.79 | Case Management | Delivery |
| 37 | What are the costs and benefits of the education measures to decrease diarrhoeal disease in the developing world? | 81.40 | 0.63 | Emerging Interventions | Delivery |
| 38 | Integration of health education services in health service facilities of government and NGOs and assess its cost-effectiveness on developmental outcomes. | 81.33 | 0.67 | Nutrition & Long-Term Outcomes | Delivery |
| 39 | Which strategies and messages are effective in convincing health care providers of the advantage of ORS and zinc compared to antibiotics or other drugs? | 81.26 | 0.73 | Case Management | Delivery |
| 40 | Evaluate calcium-supplemented ORS to reduce fluid secretion through enterocyte calcium receptors. | 81.23 | 0.65 | Emerging Interventions | Development |
| 41 | What factors, including mothers’ education, would influence acceptability of zinc supplementation and high/earlier use of ORS in the community? | 80.96 | 0.60 | Preventive Nutrition Strategies | Delivery |
| 42 | What are the key transmission pathways and dominant pathogens of diarrhoeal disease in different settings? | 80.93 | 0.57 | WASH Interventions | Description |
| 43 | What is the sensitivity and specificity of the current home oral rehydration treatment and ORS questions in DHS and MICS and are there better questions to measure use of ORS? | 80.55 | 0.67 | Monitoring & Evaluation | Description |
| 44 | How might HWTS demonstration and ORT corners increase uptake and use of HWTS products and subsequent reductions of diarrhoeal disease incidence in mothers presenting with infants and ORT corners? | 80.54 | 0.65 | Other Innovations | Development |
| 45 | Which micronutrients or macronutrients are more effective in protecting/preventing children from enteric infections and diarrhoea early in life? | 80.51 | 0.73 | Nutrition & Long-Term Outcomes | Development |
| 46 | What is the best way to improve the microbial quality of the food served to children 0-72 months at home? | 80.50 | 0.51 | WASH Interventions | Development |
| 47 | Does continued feeding during diarrhoea reduce weight loss for diarrhoea and risk of stunting? | 80.39 | 0.70 | Other Innovations | Delivery |
| 48 | What is the effectiveness of iCCM in increasing coverage of zinc and ORS? | 80.33 | 0.64 | Case Management | Delivery |
| 49 | What are the prospects for developing new rehydration solutions for use in both developed and developing countries? | 80.32 | 0.67 | Emerging Interventions | Development |
| 50 | What is the best way to develop locally appropriate and highly nutritious diets, which are effective in diarrhoea management, given the great diversity of food available in low-income countries? | 80.23 | 0.70 | Nutrition & Long-Term Outcomes | Development |
| 51 | Certain measures of long-term impact might be more valuable for policy makers. IN particular, if they can be quantified, measures of long-term diarrhoea impact that are associated with educational outcomes can convincingly demonstrate the direct costs diarrhoea imposes on schooling systems. Many policy makers seem to be more interested in direct costs to the treasury than general measures of lower productivity. | 80.13 | 0.52 | Nutrition & Long-Term Outcomes | Delivery |
| 52 | Which BCC/IEC messages are most effective in increasing the percentage of caregivers receiving zinc and ORS for children in the treatment of diarrhoea? | 80.03 | 0.65 | Other Innovations | Delivery |
| 53 | What micronutrient deficiencies are risk factors for diarrhoea incidence or severity? | 79.74 | 0.61 | Disease Burden, Aetiology & Distribution | Description |
| 54 | How best to test probiotics as an adjuvant to enhance vaccine success (e.g. in a setting where efficacy of rotavirus vaccine is not fully satisfactory)? | 79.72 | 0.63 | Emerging Interventions | Development |
| 55 | How does childhood diarrhoeal illness correlate with adult height? What is the impact of acute, prolonged, persistent and recurrent diarrhoea on growth trajectories of children in impoverished endemic areas? | 79.70 | 0.65 | Nutrition & Long-Term Outcomes | Description |
| 56 | Assess effectiveness of delivery strategies to provide zinc and ORS. | 79.67 | 0.76 | Case Management | Delivery |
| 57 | What are the barriers to continued feeding by caregivers during diarrhoea episodes? | 79.58 | 0.66 | Other Innovations | Development |
| 58 | Is the current way to measure zinc coverage in household surveys the most appropriate one? Is there a way we could measure this better? | 79.54 | 0.66 | Monitoring & Evaluation | Delivery |
| 59 | How do nutritional imbalances affect the frequency and quantity of acute diarrhoeal disease? | 79.47 | 0.66 | Emerging Interventions | Description |
| 60 | Awareness raising programs using media involvement (posters, banners, drama, radio, TV, flip charts, etc.) to combat/prevent diarrhoea and effects of childhood cognition and cost analysis. | 79.46 | 0.65 | Nutrition & Long-Term Outcomes | Development |
| 61 | How best to implement what is now available: vaccines, oral rehydration, referral algorithm? | 79.15 | 0.63 | Emerging Interventions | Delivery |
| 62 | What would be the long-term costs to human capital of preventing diarrhoea mortality without preventing diarrhoea morbidity? | 79.15 | 0.51 | Nutrition & Long-Term Outcomes | Description |
| 63 | Assess the efficacy of zinc supplementation as an adjunct to standard anti-Shigella treatment on the guy mucosal and systemic response. | 78.96 | 0.75 | Case Management | Development |
| 64 | Do responsive feeding interventions impact diarrhoeal disease outcomes? | 78.93 | 0.62 | Nutrition & Long-Term Outcomes | Development |
| 65 | Develop a successful vaccine against Cryptosporidium, Shigella, Giardia. | 78.88 | 0.55 | Nutrition & Long-Term Outcomes | Discovery |
| 66 | What is the acceptability/adherence for zinc supplementation for the management of diarrhoea in various settings (urban, rural)? | 78.83 | 0.73 | Case Management | Development |
| 67 | What are the factors driving inappropriate use of antibiotics and other inappropriate drugs by health care practitioners after over 25 years of global guidelines, national policy, training and available information; and what actions can effectively modify those inappropriate practices? | 78.63 | 0.68 | Case Management | Development |
| 68 | Research suggests that cognitive deficits associated with early childhood diarrhoea persist for at least 4 or 5 years. What measures can capture this deficit? How long does this deficit persist?^[[2]](#footnote-2)^ | 78.57 | 0.69 | Nutrition & Long-Term Outcomes | Discovery |
| 69 | What are the environmental and social/behavioural risk factors for diarrhoea? | 78.48 | 0.69 | Disease Burden, Aetiology & Distribution | Description |
| 70 | What are the fully burdened cost-benefits of different diagnostics technologies – molecular vs. culture vs. antigen detection vs. microscopy? | 78.31 | 0.59 | Diagnostics | Discovery |
| 71 | What additional impact on household diarrhoea management do other communication strategies have (“champions,” community leaders, BRAC ‘Tupperware’ model, entertainment education, billboards)? | 78.26 | 0.50 | Monitoring & Evaluation | Delivery |
| 72 | To what extent does the connection between childhood diarrhoea and undernutrition contribute to lethal comorbidities such as pneumonia, malaria and TB? | 78.22 | 0.73 | Nutrition & Long-Term Outcomes | Description |
| 73 | What is the role of short-chain fatty acid delivery to the colon in enhancing sodium and water absorption, reducing fluid secretion and facilitating mucosal repair? | 77.98 | 0.61 | Emerging Interventions | Discovery |
| 74 | Evaluate and address the systemic barriers to distribution of zinc through non-pharmaceutical shops and community health workers. | 77.90 | 0.53 | Other Innovations | Delivery |
| 75 | Develop definitive clinical trials to delineate the efficacy of “super” ORS (glucose-based ORS, cereal-based ORS and rice-based ORS). | 77.88 | 0.69 | Emerging Interventions | Development |
| 76 | What is the best current estimate of child mortality from diarrhoea globally and in various regions of the world? | 77.88 | 0.63 | Disease Burden, Aetiology & Distribution | Description |
| 77 | Does hand washing promotion during the perinatal period, which may be a unique teachable moment, lead to lasting changes in maternal hand washing behaviour and diarrhoea risk reduction? | 77.84 | 0.65 | Other Innovations | Development |
| 78 | Why do some children with acute diarrhoea go on to develop chronic diarrhoea and what can be done to prevent this? | 77.80 | 0.66 | Emerging Interventions | Description |
| 79 | What are the diarrhoeal organisms (bacteria, protozoa, virus, parasite) that affect cognition? Do specific pathogens pose a higher risk for adverse long0term outcomes? Which enteric pathogen is more associated with the worst cognitive deficit, after controlling for the same confounder in children? | 77.67 | 0.75 | Nutrition & Long-Term Outcomes | Description |
| 80 | What is the role of enteric infections in stunting? | 77.45 | 0.59 | Emerging Interventions | Description |
| 81 | Do curative-focused messages adversely influence preventative messages and adherence to 10 days of treatment? | 77.37 | 0.50 | Monitoring & Evaluation | Delivery |
| 82 | What is the impact of intermittent water supple on diarrhoeal disease and how can we ensure the microbiological quality of intermittent piped supply? | 77.05 | 0.57 | WASH Interventions | Development |
| 83 | By how much does diarrhoea (by whatever severity measure and timing we find to be most associated with developmental deficits) increase the likelihood of class repetition (worth a full pupil year of schooling as well as the infrastructure costs associated with larger classes)? By how much does diarrhoea increase the likelihood of drop-out (worth several pupil years of schooling)? | 76.97 | 0.53 | Nutrition & Long-Term Outcomes | Description |
| 84 | To know which pathogen is the most important cause of diarrhoea in target ages, seasons and regions. | 76.95 | 0.70 | Diagnostics | Description |
| 85 | To what extent do diarrhoeal illnesses or other enteric infections contribute to neurodevelopmental deficits independently of their effect on nutritional status? | 76.95 | 0.69 | Nutrition & Long-Term Outcomes | Description |
| 86 | Which clinical trials should be supported to test calcium-supplemented ORS to reduce fluid secretion through enterocyte calcium receptors? | 76.93 | 0.60 | Emerging Interventions | Development |
| 87 | Testing of locally adapted health messages to promote zinc and to explain zinc to mothers/users. | 76.85 | 0.73 | Case Management | Delivery |
| 88 | Develop age-appropriate, geography-appropriate, duration-appropriate (acute/chronic), and characteristic-appropriate (bloody/non-bloody) algorithms for management of different diarrhoea syndromes in different paediatric hosts. | 76.83 | 0.57 | Diagnostics | Development |
| 89 | What is the effect of intermittent therapy with zinc on diarrhoea prevention when given at routine contacts? | 76.77 | 0.69 | Preventive Nutrition Strategies | Development |
| 90 | What measures of diarrhoea severity are most associated with long-term developmental delays and cognitive deficits (frequency of episodes, duration, weight-loss, etc.), and how great are these? Is there a cut-off for the number of days for diarrhoeal illnesses in the first 2 years of life that can affect future development? | 76.76 | 0.69 | Nutrition & Long-Term Outcomes | Description |
| 91 | Determine which elements of planned diarrhoea control activities are actually executed and why. E.g. how does structure for program coordination affect implementation and sustainability of programs? | 76.76 | 0.52 | Monitoring & Evaluation | Description |
| 92 | What are the major bacterial, viral and parasitic pathogens responsible for mortality/morbidity in acute and chronic diarrhoea among children worldwide? Are there global monitoring systems? | 76.69 | 0.68 | Diagnostics | Description |
| 93 | Do private sector providers need training or is orientation enough? If training is required, what would be the content, who would do it and the format? | 76.68 | 0.56 | Monitoring & Evaluation | Delivery |
| 94 | What are optimal trajectories of growth in early childhood to ensure adequate cognitive development but avoid long-term consequences related to metabolism/obesity? | 76.67 | 0.63 | Nutrition & Long-Term Outcomes | Description |
| 95 | What are the most appropriate measures of equity that can be universally applied to national diarrhoea management scale up programs? | 76.62 | 0.54 | Monitoring & Evaluation | Description |
| 96 | What types of diets are more adequate for catch-up growth and better recovery after intestinal barrier disruption? | 76.53 | 0.61 | Nutrition & Long-Term Outcomes | Development |
| 97 | Identify the determinants of low use of ORS/zinc among children in target populations and evaluate strategies to modify those determinants in order to increase appropriate treatment. | 76.52 | 0.71 | Case Management | Description |
| 98 | What is the effect ton use and coverage of new delivery strategies for ORS and zinc (i.e. promotion of DTK, sales of ORS and zinc by the commercial/non-medical sector, etc.)? | 76.46 | 0.72 | Case Management | Delivery |
| 99 | Assess the optimal dose and duration of glutamine in the management of diarrhoea. | 76.38 | 0.57 | Emerging Interventions | Development |
| 100 | RCTs comparing nutritional education with anti-helminthic treatment among school aged children and assessing memory, IQ and school achievement. | 76.33 | 0.71 | Nutrition & Long-Term Outcomes | Development |
| 101 | Are the determinants of diarrhoeal deaths the same as they were in 1980 when the strategy to fight diarrhoea was developed? (If they have changed, does this change suggest new interventions to further reduce mortality)? | 76.33 | 0.62 | Emerging Interventions | Description |
| 102 | Determine the most appropriate indicators to measure the correct administration of antibiotics for diarrhoea in community settings. | 76.29 | 0.57 | Monitoring & Evaluation | Delivery |
| 103 | Assessment of key knowledge gaps in community awareness of the relationship between nutrition and the occurrence of diarrhoea and the relationship between diarrhoea and long-term development in children. | 76.29 | 0.54 | Preventive Nutrition Strategies | Delivery |
| 104 | What is the potential for inclusion of water purification tabs, pre-mixed ORS, or other clean water solutions (co-packaged with zinc) to improve treatment of paediatric diarrhoea? | 76.28 | 0.54 | Other Innovations | Delivery |
| 105 | Can caregivers be convinced to purchase zinc and ORS in advance, when it might expire before it is used? (Evaluation of “buy in advance messages”) (zinc shelf life is several years) | 76.09 | 0.71 | Case Management | Delivery |
| 106 | What are the triggers of hand washing behaviour change at different occasions and for different target groups, e.g. parents, adolescents? | 76.09 | 0.55 | WASH Interventions | Development |
| 107 | Which clinical trials should be undertaken to evaluate the efficacy and safety of anti-secretory drugs (given along with ORS and zinc supplementation) on diarrhoea stool output? | 76.02 | 0.55 | Emerging Interventions | Development |
| 108 | Assess cost effectiveness of outpatient treatment of shigellosis. | 75.71 | 0.67 | Case Management | Delivery |
| 109 | Determine how to best measure, document and use morbidity and mortality reduction attributable to specific diarrhoea control interventions (prevention and treatment) for program improvement. | 75.59 | 0.50 | Monitoring & Evaluation | Description |
| 110 | Define ways to reduce duration of antimicrobial therapy for dysentery, including single-dose therapy, through selection of appropriate agents. | 75.56 | 0.72 | Case Management | Development |
| 111 | What is the cost-effectiveness of water, sanitation and hand washing interventions for diarrhoea prevention? | 75.46 | 0.58 | Other Innovations | Delivery |
| 112 | How to develop and evaluate the safety and efficacy of anti-secretory drugs (e.g. enkephalinase inhibitors and anti-CFTR) as therapy of enterotoxin-mediated secretory diarrhoeas? | 75.40 | 0.61 | Emerging Interventions | Development |
| 113 | What are markers of malnutrition that precede growth faltering during infancy? | 75.34 | 0.58 | Nutrition & Long-Term Outcomes | Description |
| 114 | What clinical trials should be promoted to evaluate the efficacy of short-chain fatty acids (or precursors) on stool output? | 75.27 | 0.60 | Emerging Interventions | Development |
| 115 | Development of a monitoring and evaluation toolkit to document the effectiveness/impact of private commercial systems in distributing and selling ORS and zinc, taking maximum advantage of OTC status. What is the reach of distribution networks, their profitability and sustainability? | 75.22 | 0.48 | Monitoring & Evaluation | Delivery |
| 116 | What effect does the provision of sanitation and water supply in schools have on community behaviours with respect to sanitation and hygiene and what are the health outcomes for children in school and for the wider community? | 75.15 | 0.54 | WASH Interventions | Development |
| 117 | Identify formulation that increases acceptability of zinc (e.g. taste, convenience). | 75.11 | 0.73 | Case Management | Development |
| 118 | By what mechanisms (at gut and immunologic level) do malnutrition and various micronutrient deficiencies increase risk of severe diarrhoea? | 74.94 | 0.91 | Disease Burden, Aetiology & Distribution | Discovery |
| 119 | Develop an effective and safe ORS with improved taste. | 74.91 | 0.61 | Emerging Interventions | Development |
| 120 | Identify and validate biomarkers of ‘gut health’ (e.g. gut barrier function, inflammatory biomarkers, etc.) to identify those children at risk of chronic enteropathy. | 74.81 | 0.58 | Emerging Interventions | Discovery |
| 121 | How do we scale up manufacturing and distribution of co-packaged zinc and ORS packs? | 74.80 | 0.53 | Other Innovations | Development |
| 122 | How can we utilize data collected on childhood diarrhoeal diseases to reduce rates of infection and disease? Can this data be used to help target the development of specific vaccines, or will vaccines actually be applicable? On the other hand, can these data be used to target areas for improved hygiene/sanitation to reduce incidence? | 74.76 | 0.56 | Diagnostics | Description |
| 123 | What is the potential for women’s groups or peer-counselling/training of community-based cadres to improve infant/child nutrition and reduce diarrhoea through the uptake of preventive/therapeutic strategies? | 73.73 | 0.60 | Preventive Nutrition Strategies | Development |
| 124 | What is the role of Vitamin D deficiency in diarrhoea risk? | 74.61 | 0.56 | Other Innovations | Discovery |
| 125 | How can household surveys most accurately measure compliance to recommended protocols? | 74.51 | 0.55 | Monitoring & Evaluation | Delivery |
| 126 | Is it possible to use cross-sectional surveys to assess diarrhoea incidence or prevalence? | 74.50 | 0.89 | Disease Burden, Aetiology & Distribution | Description |
| 127 | Determine which approaches by global actors/health initiatives, donors and NGOs will most effectively influence policy makers and program managers to prioritize childhood diarrhoea control programs. | 74.50 | 0.52 | Monitoring & Evaluation | Development |
| 128 | Why is childhood diarrhoea mortality declining in many countries? | 74.46 | 0.59 | Disease Burden, Aetiology & Distribution | Description |
| 129 | Study the effect of zinc on the gut secretory mechanisms. | 74.42 | 0.71 | Case Management | Discovery |
| 130 | Clinical trials to evaluate the efficacy of zinc among older children and adults. This data might be critical to improve the private sector marketplace by expanding the target population. | 74.39 | 0.75 | Case Management | Development |
| 131 | Develop and test an ordering algorithm for health worker/community workers/physicians for identifying causative agent of diarrhoea in an individual or outbreak situation (diagnostic test ordering algorithm). | 74.35 | 0.62 | Diagnostics | Description |
| 132 | What is the effect of promoting flavoured ORS for home use on use and coverage, when compared to the promotion of standard ORS? | 74.34 | 0.71 | Case Management | Delivery |
| 133 | Determine whether iron and other micronutrient supplements reduce the effectiveness of zinc to prevent diarrhoeal disease (RCTs). | 74.23 | 0.66 | Preventive Nutrition Strategies | Development |
| 134 | To what extent does the simultaneous national scale up of other micronutrients for example multiple micronutrient powders, influence the scale up of ORS and zinc? Can synergies be found? | 74.17 | 0.51 | Monitoring & Evaluation | Delivery |
| 135 | Identify the determinants that result in children failing to receive ORS. | 74.02 | 0.70 | Case Management | Description |
| 136 | Does ‘preposition’/’sampling’ platform (e.g. Child Health Weeks) result in caregivers seeking ORS and zinc for future episodes once supply in gone? | 74.00 | 0.71 | Case Management | Delivery |
| 137 | What is the impact of different bundles of interventions on diarrhoea mortality in low to high diarrhoea mortality burden populations/countries? | 73.92 | 0.54 | Monitoring & Evaluation | Delivery |
| 138 | Which are the most sustainable, affordable and acceptable latrine options for the poor? | 73.90 | 0.57 | WASH Interventions | Development |
| 139 | What are the safety and efficacy of probiotics and functional foods in settings of acute diarrhoea? | 73.85 | 0.63 | Emerging Interventions | Development |
| 140 | Assess cost effectiveness of inpatient treatment of diarrhoea with ORS and zinc. | 73.80 | 0.65 | Case Management | Delivery |
| 141 | Is maternal responsiveness a protective factor for reducing episodes of diarrhoeal disease? | 73.79 | 0.61 | Nutrition & Long-Term Outcomes | Description |
| 142 | Can complementary foods be adequately fortified with micronutrients that help reduce diarrhoea? | 73.72 | 0.65 | Preventive Nutrition Strategies | Development |
| 143 | Assess effectiveness of programmers introducing ORS and zinc in the treatment of diarrhoea in the community on cost of treatment, access to treatment, ORS use rates, antimicrobial use rates, hospital visits for subsequent morbidity, case fatality rates, physicians prescription rates. | 73.67 | 0.68 | Case Management | Delivery |
| 144 | RCT comparing education on psychosocial stimulation during recovery phase of persistent diarrhoea with other educational health and hygiene packages and control looking at effects on child development at early age. | 73.62 | 0.64 | Nutrition & Long-Term Outcomes | Development |
| 145 | What are the pathogens associated with acute and persistent diarrhoea in world regions? | 73.62 | 0.60 | Disease Burden, Aetiology & Distribution | Description |
| 146 | What is the most effective mix of WASH interventions in different epidemiological settings? | 73.58 | 0.50 | WASH Interventions | Development |
| 147 | How can we truly measure willingness vs. ability to pay? | 73.51 | 0.55 | Monitoring & Evaluation | Description |
| 148 | How can the private sector (including informal sector) be used to increase coverage of zinc treatment of diarrhoea? | 73.47 | 0.68 | Case Management | Delivery |
| 150 | Can the rotavirus vaccine introduction be used as an opportunity to increase focus on and resources for a comprehensive package of diarrhoea control? | 73.31 | 0.47 | Monitoring & Evaluation | Delivery |
| 151 | What are the sources and mechanisms of spread of childhood diarrhoeal illness that are most associated with poor cognitive outcomes? What are the knowledge, attitude, belief, practice and limitations to sanitary facilities associated with frequency of childhood diarrhoea and their association with cognition? | 73.22 | 0.63 | Nutrition & Long-Term Outcomes | Description |
| 152 | What is the efficacy of a combination of probiotics, such as *Sacchoromyces boulardii* and LGG, for preventions and treatment of acute diarrhoea in the developing world? | 73.21 | 0.62 | Emerging Interventions | Development |
| 153 | Given the higher number of over-the-counter medicines available in lower income countries, including antibiotics, what is the role of these medications in prolongation of diarrhoea and effect on nutritional and long-term outcomes? | 73.17 | 0.55 | Nutrition & Long-Term Outcomes | Description |
| 154 | How best to identify and change the determinants of HWWS behaviours so as to get effective behaviour changes? | 73.17 | 0.44 | WASH Interventions | Delivery |
| 155 | What is the impact of water collection time (time to source) on diarrhoea? | 73.06 | 0.55 | WASH Interventions | Description |
| 156 | How best to dispose of child faeces and what is their health impact? | 73.01 | 0.51 | WASH Interventions | Development |
| 157 | Can a water storage vehicle be developed with slow release halogen? | 72.77 | 0.53 | Other Innovations | Discovery |
| 158 | Can the private and public sector co-exist to co-promote proper treatment and can these two work together to increase coverage of ORS and zinc? | 72.75 | 0.62 | Case Management | Delivery |
| 159 | What is the safety and efficacy of targeting single-dose chemotherapy (as perhaps with albendazole, azithromycin, and possibly nitazoxanide; the first 2 already being “rolled out”) in improving growth trajectories (as well as diarrhoea – and even other illness – rates)? | 72.71 | 0.64 | Nutrition & Long-Term Outcomes | Development |
| 160 | Identify and test education or other interventions to reduce microbial contamination in weaning and complementary foods. | 72.70 | 0.66 | Preventive Nutrition Strategies | Development |
| 161 | Can and should cheap and rapid diagnostic tests for common enteric pathogens be created for use in the field?   1. Will field or clinic use of rapid diagnostic test for common enteric infections lead to improved accuracy of disease diagnosis (and more appropriate, targeted therapies or health measures) 2. Will more rapid and accurate diagnosis of enteric diseases (and use of targeted therapies improve measures of disease outcome and long term health? How to measure?   Will targeted therapies and new diagnostics decrease antibiotic resistance? | 72.70 | 0.58 | Diagnostics | Discovery |
| 162 | How do potential gains in learning from treating diarrhoea compare with the effectiveness of other quality improvement efforts (maybe room for AKU-IED to give some data on costs associated with learning gains associated with their programs, or programs run by the government and other aid agencies)? | 72.70 | 0.49 | Nutrition & Long-Term Outcomes | Development |
| 163 | Determine what are the essential elements of, and how best to use, public-private partnership for diarrhoea disease control. | 72.66 | 0.52 | Monitoring & Evaluation | Description |
| 164 | Research to evaluate the impact of low birth weight (LBW) to subsequent malnutrition and susceptibility to diarrhoeal disease among infants and young children. | 72.65 | 0.60 | Preventive Nutrition Strategies | Description |
| 165 | What is the effect of enteral glutamine on mucosal and systemic immune responses in children with diarrhoea? | 72.65 | 0.60 | Emerging Interventions | Discovery |
| 166 | Identify the determinants that result in children failing to receive zinc. | 72.64 | 0.70 | Case Management | Delivery |
| 167 | Assess cost effectiveness of outpatient treatment of diarrhoea with zinc and ORS. | 72.62 | 0.65 | Case Management | Delivery |
| 168 | What is the health impact of different levels, and types of sanitation coverage? | 72.62 | 0.57 | WASH Interventions | Description |
| 169 | What is the incidence and prevalence of acute (without and with dehydration) diarrhoea and persistent diarrhoea in urban and rural populations and by SES? | 72.60 | 0.63 | Disease Burden, Aetiology & Distribution | Description |
| 170 | How do missed opportunities in childhood development affect the global burden of diarrhoeal disease? | 72.58 | 0.49 | Nutrition & Long-Term Outcomes | Description |
| 171 | What explains the recent declines in diarrhoea mortality globally? | 72.46 | 0.51 | WASH Interventions | Description |
| 172 | What is the effect of routine use of preventive community supplementation of zinc on incidence and severity of diarrhoea in <36 months with and without Vitamin A supplements? | 72.44 | 0.65 | Preventive Nutrition Strategies | Development |
| 173 | What does it take to sustain hygiene behaviour change? And what is the role of norms? | 72.42 | 0.55 | WASH Interventions | Development |
| 174 | Define “scale” when it comes to coverage of recommended interventions for childhood diarrhoea programs and develop standard metrics for scale-up. | 72.34 | 0.46 | Monitoring & Evaluation | Description |
| 175 | Efficacy, effectiveness and cost studies that will increase the utilization of zinc food fortification programs in developing countries. | 72.30 | 0.57 | Preventive Nutrition Strategies | Development |
| 176 | What is the efficacy of selected recommended home fluids (i.e. rice water solution/other) for the prevention of dehydration? | 72.29 | 0.73 | Case Management | Development |
| 177 | What more can be done to improve administration of ORS in terms of taste and volume of intake (something currently being explored by Medentech)? | 72.26 | 0.54 | Other Innovations | Development |
| 178 | Assess the utility of targeting NKCC, K channels and Na-coupled transporters in diarrhoea therapy. | 72.19 | 0.58 | Emerging Interventions | Discovery |
| 179 | To what extent are community water treatment interventions more effective in reducing diarrhoeal disease incidence than promotion of HWTS interventions? | 72.12 | 0.55 | Other Innovations | Development |
| 180 | How can marketing approaches contribute to and sustain sanitation? | 72.00 | 0.53 | WASH Interventions | Delivery |
| 181 | How can WASH interventions be scaled up and targeted to those who most need it? | 71.94 | 0.47 | WASH Interventions | Delivery |
| 182 | What is the impact of IMCI in different population groups on administration and promotion of zinc therapy for acute and persistent diarrhoeas? | 71.90 | 0.63 | Case Management | Delivery |
| 183 | Does underlying vulnerability (e.g. undernutrition, poor access to treatment) affect disparities in the WASH-related diarrhoea burden? | 71.87 | 0.52 | WASH Interventions | Description |
| 184 | What point-of-care diagnostics will strengthen both patient care and diarrhoea surveillance? | 71.77 | 0.54 | Monitoring & Evaluation | Description |
| 185 | What are the enteric pathogens most commonly associated with diarrhoea among HIV-infected persons? | 71.61 | 0.60 | Disease Burden, Aetiology & Distribution | Description |
| 186 | What is the added valued of diagnostics for enterics from a public health cost standpoint: are certain pathogens more important to know about for prognostic/clinical reasons and what are those scenarios? | 71.51 | 0.58 | Diagnostics | Description |
| 187 | Among developing countries, which three have achieved the highest national usage rates for zinc and ORS in the treatment of paediatric diarrhoea – and what strategies/interventions were most effective in increasing usage? | 71.49 | 0.54 | Other Innovations | Description |
| 188 | Determine how the following elements influence the effectiveness and sustainability of national diarrhoea control programs:   1. National leadership, public sector structure and funding, and multi-sectoral participation in planning and coordination 2. Health management information systems (determinants, coverage of safe water and sanitation, morbidity, mortality, etc.) | 71.39 | 0.47 | Monitoring & Evaluation | Description |
| 189 | Evaluation of the effectiveness of educational reminders through mobile phones, jingles or other media marketing mechanisms as reinforcement of early use of fluid in diarrhoea, increased fluids, continued nutrition and breast feeding promotion. | 71.39 | 0.62 | Preventive Nutrition Strategies | Delivery |
| 190 | Is zinc in fortified foods as effective as zinc supplements in the prevention of diarrhoea? | 71.32 | 0.72 | Preventive Nutrition Strategies | Development |
| 191 | What is the impact of intermittent water supple on diarrhoea? | 71.23 | 0.55 | WASH Interventions | Description |
| 192 | Does the roll-out of rotavirus vaccination change the distribution of rotavirus genotypes in children? | 71.02 | 0.72 | Disease Burden, Aetiology & Distribution | Description |
| 193 | Clinical trial to evaluate the efficacy and effectiveness of the new anti-cryptosporidial drugs/agents. | 70.98 | 0.68 | Case Management | Development |
| 194 | What is the better approach to improve the intestinal microbiome in order to enrich the nutrient absorption and protect the intestinal barrier function following enteric infections? | 70.95 | 0.51 | Nutrition & Long-Term Outcomes | Discovery |
| 195 | What is the importance of environmental enteropathy relative to diarrhoeal disease for child health? | 70.95 | 0.49 | WASH Interventions | Description |
| 196 | What is the most effective training method for different cadres of health care workers in the public and private sectors? | 70.88 | 0.59 | Case Management | Delivery |
| 197 | What is the impact of lactose intolerance and food allergy following acute diarrhoea? | 70.81 | 0.56 | Emerging Interventions | Description |
| 198 | How might promotion of durable water filters with a financing mechanism for purchase sold through “basket of goods” business models increase uptake and use across all HWTS products, and subsequent reduction of diarrhoeal disease? | 70.66 | 0.54 | Other Innovations | Delivery |
| 199 | Has the incidence of diarrhoea decreased and if so, what factors area associated with this decrease? | 70.64 | 0.59 | Disease Burden, Aetiology & Distribution | Description |
| 200 | What pathogens are associated with fatal childhood diarrhoea? | 70.62 | 0.56 | Disease Burden, Aetiology & Distribution | Description |
| 201 | Clinical trials to evaluate the efficacy of short chain fatty acids (or its precursors) mixed with ORS and zinc supplementation on stool output in cholera and acute non-cholera diarrhoea. | 70.59 | 0.64 | Case Management | Development |
| 202 | Are there enteropathogens (particularly chronic infections for which treatment exists, i.e. helminths) that modulate the incidence and severity of other enteropathogens? | 70.59 | 0.55 | Disease Burden, Aetiology & Distribution | Discovery |
| 203 | How can the introduction of rotavirus vaccine be used to also increase appropriate treatment of children with diarrhoea? | 70.41 | 0.63 | Case Management | Delivery |
| 204 | What is the health impact of large scale WASH behavioural interventions? | 70.41 | 0.47 | WASH Interventions | Development |
| 205 | Where multiple deficiencies co-exist, what is the relative efficacy (and safety) of preventive supplementation with zinc along compared to preventive supplementation with zinc in combination with other micronutrients/? What is the optimal dosing for zinc? | 70.36 | 0.68 | Preventive Nutrition Strategies | Development |
| 206 | How much of diarrhoea mortality is from watery diarrhoea versus dysentery versus persistent diarrhoea? | 70.35 | 0.61 | Disease Burden, Aetiology & Distribution | Description |
| 207 | What threshold of infection (by pathogen virulence, burden of diarrhoea, etc.) could cause an irreversible kind of brain injury in children? Does this threshold change with or without undernutrition? | 70.31 | 0.59 | Nutrition & Long-Term Outcomes | Description |
| 208 | Although mucosal immune responses are elicited by oral vaccines, responses to such vaccines may be of relatively short duration compared to that induced by wild type disease. What are the reasons for this? What are the mediators and modifiers of long-term mucosal immunity? Would improved vaccines, regimens and/or immunization strategies result in longer duration? *V. cholerae* is a well studied, paradigmatic, non-invasive pathogen, and oral killed and live attenuated cholera vaccines, as well as subunit and conjugate vaccines exist. Could cholera be used as a mucosal model to address these questions? Such an approach may not only inform how to induce long-term immunity against mucosal pathogens as a group, but, if successful, could facilitate roll out and implementation of current or improved cholera vaccines. | 70.24 | 0.71 | Vaccines | Discovery |
| 209 | In randomized controlled field trials in sub-Saharan Africa and South and Southeast Asia, oral rotavirus vaccines have conferred ~50-60% efficacy. WHO SAGE has recommended their use and GAVI has committed to finance introduction of rotavirus vaccine into national EPIs. If a poor sub-Saharan African country achieves a high coverage of rotavirus vaccines, is it conceivable that the indirect protective effects, in addition to the direct protection, may result in a greater than expected impact on diminishing disease burden? Should it be a high priority to affirm (or disprove) this hypothesis since it has important public health implications? | 70.18 | 0.66 | Vaccines | Development |
| 210 | Could dietary ‘errors’ contribute to transforming acute diarrhoea intro chronic diarrhoea? | 69.99 | 0.53 | Emerging Interventions | Description |
| 211 | Efficiently and effectively screen local herbal remedies for diarrhoea (e.g. efficacy in relevant animal models and on specific targets, such as chloride channels) | 69.97 | 0.55 | Emerging Intervention | Development |
| 212 | Assess cost-effectiveness of inpatient treatment of severe shigellosis. | 69.93 | 0.68 | Case Management | Delivery |
| 213 | How or should health provider childhood diarrhoea management promotion change by type of provider? | 69.85 | 0.44 | Monitoring & Evaluation | Delivery |
| 214 | Is there a sufficient evidence base to provide delivery model guidelines for the planning of national diarrhoea scale up initiatives? | 69.83 | 0.46 | Monitoring & Evaluation | Delivery |
| 215 | What proportion of deaths from other causes also have diarrhoea as a comorbidity (i.e. is diarrhoea a risk factor for death from other causes)? | 69.82 | 0.59 | Disease Burden, Aetiology & Distribution | Description |
| 216 | What are the critical control points in the delivery system that are barriers to full and effective integration of childhood diarrhoea management and prevention within child health and nutrition programs? | 69.79 | 0.49 | Monitoring & Evaluation | Delivery |
| 217 | What is the role of probiotic agents in improving management of severe malnutrition and diarrhoea? | 69.77 | 0.53 | Other Innovations | Development |
| 218 | What clinical trials should be undertaken o evaluate the efficacy and safety of probiotic-derived antibacterial synthetic peptides on diarrhoeal disease? | 69.69 | 0.55 | Emerging Interventions | Development |
| 219 | Do mystery client surveys provide any added value to the monitoring of provider diarrhoea treatment practices? | 69.69 | 0.49 | Monitoring & Evaluation | Description |
| 220 | Research to develop interventions for low birth weight (LBW) to prevent the subsequent malnutrition and susceptibility to diarrhoeal disease among infants and young children. | 69.60 | 0.51 | Preventive Nutrition Strategies | Development |
| 221 | To assess currently available tests for their sensitivity, specificity, speed and cost for diagnoses of diarrhoea. | 69.56 | 0.65 | Diagnostics | Description |
| 222 | What is the importance of animal faeces in diarrhoeal disease? | 69.53 | 0.50 | WASH Interventions | Description |
| 223 | Assess the impact of alleviation of food insecurity on the burden of diarrhoeal disease. | 69.52 | 0.50 | Preventive Nutrition Strategies | Development |
| 224 | What market-based incentives can be established to increase marketing efforts by manufacturers of zinc and ORS? | 69.35 | 0.51 | Other Innovations | Delivery |
| 225 | What should be the content of community health volunteer or worker training and follow-up support? How to best integrate training into overall iCCM training? | 69.30 | 0.46 | Monitoring & Evaluation | Delivery |
| 226 | Clinical trials to compare the efficacy of different available low cost drugs for the management of shigellosis. | 69.29 | 0.67 | Case Management | Development |
| 227 | How can we best build the documentation base and capture data on models that are working, common barriers, cost-effectiveness, etc.? | 69.05 | 0.51 | Monitoring & Evaluation | Delivery |
| 228 | Investigate: Are there differences in perception of taste in terms of acceptability of zinc in various cultural settings? How does taste affect acceptability of zinc? (e.g. in some setting, is bitter better/not inferior, assuring parents of medicinal value)? | 69.04 | 0.53 | Other Innovations | Development |
| 229 | Development of novel rehabilitation diets, using F75 as a basis, with different compositions (e.g. carbohydrate context, micronutrients) or Ready-to-Use-Therapeutic-Foods (RUTFs) for malnourished children with a specific aim to prevent diarrhoea. | 69.01 | 0.49 | Preventive Nutrition Strategies | Development |
| 230 | How does sustained faecal ingestion and/or repeated bouts of diarrhoea influence a child’s development and immune system? | 68.88 | 0.44 | WASH Interventions | Description |
| 231 | What are the most effective strategies to increase the percentage of private sector providers and retailers recommending/prescribing zinc and ORS for paediatric diarrhoea? | 68.70 | 0.53 | Other Innovations | Delivery |
| 232 | Clinical trials to evaluate the efficacy and safety of anti-secretory drugs given along with ORS and zinc supplementation on diarrhoeal stool output. | 68.56 | 0.60 | Case Management | Development |
| 233 | Is the quality of the care-giving environment a protective factor for reducing episodes of diarrhoeal disease? | 68.50 | 0.63 | Nutrition & Long-Term Outcomes | Description |
| 234 | How can communities be supported to manage water supply sustainability? | 68.44 | 0.54 | WASH Interventions | Delivery |
| 235 | What is the role of social norms in addressing long-term sustainability of improved WASH practices? | 68.43 | 0.50 | WASH Interventions | Delivery |
| 236 | Does washing hands of infants and toddlers decrease diarrhoea risk? | 68.24 | 0.55 | Other Innovations | Description |
| 237 | Develop and test new antibiotics for the management of Shigella dysentery. | 68.17 | 0.66 | Case Management | Development |
| 238 | How might increased product choice within a focused price range for durable HWTS products encourage uptake and use of the products among low-income households, and subsequent reduction of diarrhoeal disease? | 68.06 | 0.55 | Other Innovations | Delivery |
| 239 | Does zinc supplementation prevent diarrhoeal disease in non-zinc deficient populations? | 68.05 | 0.57 | Preventive Nutrition Strategies | Development |
| 240 | What is the effectiveness of maternal (prenatal) zinc supplementation on the outcome and incidence of diarrhoea in the community? | 68.02 | 0.58 | Preventive Nutrition Strategies | Development |
| 241 | Assess the systemic, institutional, and individual barriers to zinc uptake in high diarrhoea-burden countries. | 67.93 | 0.50 | Other Innovations | Delivery |
| 242 | How to avert stunting by diagnosis and treatment? | 67.71 | 0.44 | Emerging Interventions | Development |
| 243 | What actions are required to assure that zinc treatment of children with diarrhoea is accompanied by ORS? | 67.64 | 0.58 | Case Management | Delivery |
| 244 | Do home fluid programs or cereal based ORS programs reach more people and lead to reduced burden of diarrhoeal disease? | 67.64 | 0.48 | Preventive Nutrition Strategies | Delivery |
| 245 | What is the role of various prebiotic mixtures in preventing and treating acute diarrhoea in children? | 67.62 | 0.60 | Emerging Interventions | Development |
| 246 | What is the role of nutritional (micronutrient) interventions for diarrhoeal disease in subpopulations (i.e. HIV positive/exposed)? | 67.59 | 0.64 | Preventive Nutrition Strategies | Descrption |
| 247 | What is the incidence of multi-resistant Shigella infection among children <5y, in countries with and without high burden of HIV/AIDS? | 67.45 | 0.60 | Disease Burden, Aetiology & Distribution | Description |
| 248 | Develop molecular techniques for understanding co-infections (bacterial and parasitic and viral causative agents). | 67.37 | 0.53 | Diagnostics | Discovery |
| 249 | What clinical trials should be undertaken to evaluate the efficacy and effectiveness of nitazoxanide and tizoxanide (and other thiazolide anti-infective agents)? | 67.21 | 0.56 | Emerging Interventions | Development |
| 250 | Develop simple, cheap, hygienic approaches to use of expressed breast milk for working mothers; what is the effect of utilization of this intervention on the diarrhoeal disease burden? | 67.11 | 0.49 | Preventive Nutrition Strategies | Development |
| 251 | In view of clear reduce immunogenicity of oral enteric vaccines in children in developing countries, should significant resources be allocated to better understand the reasons for such finding and for development of alternative modes of delivery (modified oral delivery and/or alternative routes) for efficient immunization with enteric vaccines in these populations? | 67.05 | 0.65 | Vaccines | Development |
| 252 | Is infection with *G. lamblia* associated with any adverse health effects? | 66.98 | 0.56 | Disease Burden, Aetiology & Distribution | Description |
| 253 | Develop and test new antibiotics for the management of cholera. | 66.98 | 0.56 | Case Management | Development |
| 254 | What are the effects of probiotics on the mucosal and systemic immune responses in children with diarrhoea? | 66.89 | 0.57 | Emerging Interventions | Development |
| 255 | How do age, aetiology and severity of diarrhoea affect the integrity of the gut and subsequent growth/health? | 66.82 | 0.56 | Disease Burden, Aetiology & Distribution | Discovery |
| 256 | What is the right mix of sanitation approaches in different settings with different characteristics? (waste water treatment, CLTS, sanitation marketing, eco-san, low cost networks)? | 66.79 | 0.50 | WASH Interventions | Development |
| 257 | There are two licensed non-living oral cholera vaccines that require two doses to immunize and are useful for control of endemic disease. For control of epidemic cholera, particularly in unsettled and emergency situations, should resources be applied to complete development and achieve licensure of one or more single-dose oral cholera vaccines? | 66.72 | 0.67 | Vaccines | Development |
| 258 | How can we reduce pollution at the water point? | 66.72 | 0.46 | WASH Interventions | Delivery |
| 259 | Establish the role of chloride channels in rotavirus-induced diarrhoea and then develop and test inhibitors of calcium activated chloride channels. | 66.68 | 0.57 | Emerging Interventions | Discovery |
| 260 | Natural *Shigella* infection confers around 70-75% protection against the homologous serotype for a limited period of time (~2-3 years). This figure parallels the level of serum and antibody secreting cell (ASC) responses to natural infection. What would be a priority for investment of research resources: development of multicomponent (5-valent) vaccines which will cover the most common serotypes reaching this extent of protective efficacy in developing countries? And/or (?) discovery of common protein antigens (perhaps secreted proteins in vivo) which will cross-react with *Shigella* homologous and heterologous sera and further study their immunogenicity and potential to cross protect? | 66.63 | 0.66 | Vaccines | Development |
| 261 | How do aspects of the diet, particularly resistant starches, affect duration of diarrhoea? | 66.59 | 0.53 | Disease Burden, Aetiology & Distribution | Discovery |
| 262 | How best to define effective methods to impact on environmental microbe reduction? | 66.56 | 0.44 | Emerging Interventions | Description |
| 263 | Study the effect of short chain fatty acids on the mucosal secretory mechanisms. | 66.51 | 0.57 | Case Management | Discovery |
| 264 | Assess optimal dose and duration of zinc for diarrhoea treatment. | 66.47 | 0.65 | Case Management | Development |
| 265 | Develop improved markers/challenges tests for nutrient malabsorption (to determine the relative importance of different enteric infections in inducing malabsorption). | 66.43 | 0.58 | Disease Burden, Aetiology & Distribution | Discovery |
| 266 | To undertake mechanistic and intervention studies (RCTs) to determine effect of Vitamin A on (intestinal) immunology and diarrhoea. | 66.43 | 0.57 | Preventive Nutrition Strategies | Development |
| 267 | Assess the prevalence and parasite load of opportunistic intestinal protozoa (microsporidia, Cryptosporidum and Cytospora) in high and low (non) HIC endemic areas. | 66.42 | 0.63 | Diagnostics | Description |
| 268 | Develop an ideal diagnostics approach to be supported by the best available methods for microbiological confirmation of diarrhoeal agents. | 66.41 | 0.54 | Diagnostics | Description |
| 269 | Do markers of gut inflammation or intestinal injury predict linear growth deficits? | 66.35 | 0.62 | Disease Burden, Aetiology & Distribution | Description |
| 270 | Develop and improve existing algorithms for therapeutic interventions in the absence of identification of specific causative agent. | 66.32 | 0.53 | Diagnostics | Development |
| 271 | Develop and test an integrated diagnostic test that addresses the major causes of childhood febrile illness, with or without diarrhoea (pneumonia, diarrhoea, malaria). | 66.27 | 0.52 | Diagnostics | Description |
| 272 | Determine how to best use a mix of cross-sectoral approaches (e.g. health, public works/engineering, housing, public/private mix) and civil society organizations to build comprehensive and sustainable diarrhoea control programs. | 66.09 | 0.55 | Monitoring & Evaluation | Delivery |
| 273 | Among the paediatric diarrhoeal drugs under development (Crofelemer, Coloplus, others?), which are the most promising/potentially efficacious and what steps can be taken to accelerate their time to market? | 66.08 | 0.47 | Other Innovations | Development |
| 274 | Which relevant animal models should be developed to study diarrhoea pathophysiology and to test new therapies? | 66.07 | 0.52 | Emerging Interventions | Discovery |
| 275 | How much do lipid profile (serum apoproteins, HDL, LDL) and enriched fat diets effect or influence early childhood diarrhoea? Which of these factors could be associated with low/improved weight gains and poor recover/better catch-up following early childhood diarrhoea? | 66.00 | 0.58 | Nutrition & Long-Term Outcomes | Description |
| 276 | What are the best tools for monitoring the progress of WASH interventions and investment? | 65.95 | 0.48 | WASH Interventions | Delivery |
| 277 | Natural enterotoxinogenic Escherichia coli (ETEC) infection confers around 70-75% protection against the homologous strain for a limited period of time (~2-3 years). This figure parallels the level of serum and antibody secreting cell (ASC) responses to natural infection. What would be a priority for investment of research resources: development of a multicomponent (e.g. multivalent colonization factor antigen-based) vaccines which will cover the most common antigenic types reaching this extent of protective efficacy in developing countries? And/or (?) the discovery of common protein antigens (perhaps secreted proteins expressed in vivo) which will cross-react with ETEC homologous and heterologous sera and further study their immunogenicity and potential to cross protect? | 65.36 | 0.59 | Vaccines | Development |
| 278 | Develop new diagnostics for known agents. | 65.35 | 0.55 | Emerging Interventions | Discovery |
| 279 | A constellation of data from animals and humans suggest that a pentavalent Shigella vaccine consisting of S. sonnei, S. flexneri 2a, 3a, and 6 and S. dysenteriae type 1 will cover the Shigella strains of greatest clinical and epidemiologic importance. This hypothesis, known as the "CVD pentavalent strategy" is based on finding suggesting that two of the three S. flexneri serotypes (2a and 3a) slated for inclusion in the pentavalent vaccine will provide protection against the remaining 14 types and subtypes through shared group antigens. Immunization and challenge data from an animal model document the proof-of-principle of this strategy. Should selected challenge studies be performed to validate this approach in humans? | 65.33 | 0.57 | Vaccines | Development |
| 280 | Identify and define the roles of anti-inflammatory agents in the management of shigellosis and other invasive diarrhoeas. | 65.27 | 0.55 | Case Management | Discovery |
| 281 | Evaluate improved rapid assessment techniques and case mapping for cases of diarrhoea in displaced populations to prevent the spread of diarrhoeal disease within these populations. | 65.20 | 0.47 | Preventive Nutrition Strategies | Development |
| 282 | Association of APOE-4 in recovered persistent diarrhoeal (PD) and non-PD malnourished children with future cognition? | 65.03 | 0.48 | Nutrition & Long-Term Outcomes | Discovery |
| 283 | Establish mechanisms through which global ORS (and zinc) production and ORS (and zinc) sale could be monitored – e.g. testing of different approaches to allowed for triangulation and assessment of the validity of the data reported or collected. | 64.99 | 0.45 | Monitoring & Evaluation | Delivery |
| 284 | Are there adverse effects of “asymptomatic infection” by enteric pathogens? | 64.88 | 0.58 | Disease Burden, Aetiology & Distribution | Description |
| 285 | How often do “co0infections” with pathogens occur in symptomatic infants and how does this impact the accuracy of diagnosis and proper treatment? | 64.63 | 0.56 | Diagnostics | Description |
| 286 | What is the effect of a strict under 36 months growth monitoring and counselling program on reducing the burden of diarrhoea? | 64.54 | 0.59 | Preventive Nutrition Strategies | Development |
| 287 | What re the most cost effective ways to measure and share national coverage data for the percentage of children receiving zinc and ORS for the treatment of paediatric diarrhoea? | 64.48 | 0.48 | Other Innovations | Description |
| 288 | What high quality clinical trials should be undertaken to evaluate optimal constitution of intravenous rehydration fluids (i.e. dextrose content, base content, volume, rate)? | 64.46 | 0.54 | Emerging Interventions | Development |
| 289 | What impact do maternity leave policies have on breastfeeding, how do these policies affect the burden of diarrhoea? | 64.37 | 0.50 | Preventive Nutrition Strategies | Description |
| 290 | Recent studies (see below) showed that the intradermal route of delivery (in particular, the use of microneedle devices) induce stronger mucosal and cellular immune response than intramuscular, subcutaneous and transcutaneous routes when used to deliver protein antigens (influenze hemagglutinins, HIV p24 particle protein) in mice. No studies assessed the intradermal microneedle route of delivery for polysaccharides or polysaccharide-protein conjugates. Should the intradermal microneedle delivery be extensively studied for enteric antigens like Salmonella Typhi Vi, Shigella OPS-conjugates and others as a potential solution for improved immunogenicity and efficacy of these vaccines in young children in general and in developing countries in particular? Weldon et al. Clin Vaccine Immunol. 2011; Liard et al. Vaccine 20122; Koutsonanos et al. JID 20122; JK Hickling et al. Bull WHO 2011 | 64.30 | 0.64 | Vaccines | Development |
| 291 | What are the effects of probiotics on gut mucosal biology (intestinal permeability, innate and adaptive immune response and antigen trafficking)? | 64.28 | 0.54 | Emerging Interventions | Development |
| 292 | Evaluate the frequency, burden and mechanisms that are responsible for bacteremia and sepsis in children with severe diarrhoeal disease. | 64.20 | 0.46 | Preventive Nutrition Strategies | Description |
| 293 | What is the safety and cost-effectiveness of ondensetron (other 5-HT3 receptor antagonists) in children, especially for those living in LMICs? | 64.20 | 0.45 | Emerging Interventions | Delivery |
| 294 | Develop a generic platform for NAAT that is: 1) inexpensive; 2) easy to use (and probably inflexible; and, 3) closed, such that detection of the amplicons does not require opening the tubes post amplification. I thin that development of many assays would fall into place once such a system is created. | 64.19 | 0.48 | Diagnostics | Discovery |
| 295 | Develop new anti-secretory drugs (i.e. enkephalinase inhibitors, anti CFTR). | 64.08 | 0.59 | Case Management | Discovery |
| 296 | Can we develop improved early warning systems (such as train medical personnel and CHWs to identify cholera and other diseases)? | 64.03 | 0.54 | Other Innovations | Description |
| 297 | Anticipating that current vaccine development efforts will yield safe, effective and affordable ETEC and *Shigella* vaccines that both may exert a herd protective effect, how should these vaccines be given in endemic areas? For example, should ETEC be administered according to a standard EPI 3-dose schedule? Should *Shigella* be given on a modified measles schedule? Are mass vaccination days or community campaigns and option for these vaccines? | 63.73 | 0.61 | Vaccines | Development |
| 298 | Assess rapid diagnostic procedures for the detection of antibiotic multi-resistant strains of Shigella (and other enteric pathogens). | 63.39 | 0.57 | Diagnostics | Development |
| 299 | How does childhood diarrhoeal illness associate with adult cardiovascular disease and hypertension? | 63.69 | 0.57 | Nutrition & Long-Term Outcomes | Description |
| 300 | There is accumulating evidence of the negative impact of enteric diseases occurring in early childhood on the subsequent child cognitive development. Should research resources be allocated to develop vaccine cost-effectiveness calculation models, which will include this parameter as part of the burden of disease? | 63.68 | 0.61 | Vaccines | Description |
| 301 | How to delineate the roles of CFTR and calcium-activated chloride channels in diarrhoea in children? | 63.43 | 0.50 | Emerging Interventions | Discovery |
| 302 | Conduct trials to evaluate the safety and efficacy of pre- and pro-biotics given with ORS and zinc on the clinical outcome of diarrhoea. | 63.38 | 0.59 | Case Management | Development |
| 303 | Which behavioural constructs should be exploited to yield sustained improvements in household water treatment, sanitation, and hand washing behaviour at-scale? | 63.24 | 0.45 | Other Innovations | Development |
| 304 | How can the health sector be reoriented to support WASH interventions? | 63.22 | 0.47 | WASH Interventions | Delivery |
| 305 | Find appropriate vaccines, probiotics, nutritional support and improved mucosal immunity, fit the microbiome in order to prevent enteric infections and undernutrition. | 63.15 | 0.45 | Nutrition & Long-Term Outcomes | Discovery |
| 306 | How does the host microbiota impact on the frequency, severity and duration of acute diarrhoeal diseases? | 63.02 | 0.48 | Emerging Interventions | Discovery |
| 307 | What is the role of dietary fats in recovering from diarrhoea? | 62.96 | 0.50 | Emerging Interventions | Discovery |
| 308 | Improved analytic techniques (mathematical models/biostatistics) for the improved validity of attribution of pathogen to diarrhoea given most enteric infections have important asymptomatic carriage rates. | 62.95 | 0.64 | Disease Burden, Aetiology & Distribution | Description |
| 309 | Point-of-care immunocard/dip-stick test (POCT) for enterics, potentially including hand held solar powered nucleic acid detection devises. | 62.87 | 0.53 | Diagnostics | Description |
| 310 | What is the diarrhoea risk associated with water collection, transportation, storage and inconsistent use of HHWT? | 62.80 | 0.46 | WASH Interventions | Description |
| 311 | Could dietary fibres mimic the effects of prebiotics and probiotics? If so, which ones? | 62.77 | 0.46 | Emerging Interventions | Discovery |
| 312 | Which epidemiologic methods and analytic approaches are best suited to measure the association between infection with given enteropathogens and subsequent adverse outcomes of diarrhoea, including growth faltering and death? | 62.68 | 0.92 | Disease Burden, Aetiology & Distribution | Description |
| 313 | What is the incidence of unrecognized C. difficile infection in hospitalized patients in developing areas? | 62.66 | 0.53 | Diagnostics | Description |
| 314 | How does childhood diarrhoeal disease associate with asthma? | 62.59 | 0.53 | Nutrition & Long-Term Outcomes | Description |
| 315 | How can external and self-financing for WASH solutions be optimised? | 62.58 | 0.45 | WASH Interventions | Development |
| 316 | What are the factors that lead to adoption and sustained use of household water treatment? | 62.56 | 0.52 | WASH Interventions | Delivery |
| 317 | Develop low-cost latrines, such as plastic sanitation platforms (made from plastic). | 62.50 | 0.45 | Other Innovations | Development |
| 318 | Characterize symptomatic/asymptomatic disease as it affects cognition. | 62.43 | 0.55 | Nutrition & Long-Term Outcomes | Description |
| 319 | Does the widespread inappropriate use of antibiotics in developing countries contribute to the development of resistant strains infecting children in these countries? | 62.36 | 0.57 | Disease Burden, Aetiology & Distribution | Description |
| 320 | Develop rapid diagnostic procedures for the detection of antibiotic multi-resistant strains of Shigella and other bacteria. | 62.31 | 0.63 | Diagnostics | Description |
| 321 | How can the “infection-malnutrition-adverse developmental outcomes” proves be successfully modeled in animals to help understand mechanisms and prioritize the pre-clinical investigation of promising interventions? How do we “humanize” the GI tract of laboratory mice, and model dietary and environmental factors, to more adequately reflect health conditions in low-and-middle-income countries? | 62.07 | 0.48 | Nutrition & Long-Term Outcomes | Discovery |
| 322 | Do prebiotics have any preventive role to play in countries with a high burden of diarrhoeal disease? | 61.95 | 0.59 | Preventive Nutrition Strategies | Discovery |
| 323 | Conduct social marketing research to improve the acceptability of fortified foods and zinc supplements. | 61.95 | 0.47 | Preventive Nutrition Strategies | Delivery |
| 324 | What is the incidence of multi-resistant Shigella infection among children <5y in different regions? | 61.93 | 0.67 | Disease Burden, Aetiology & Distribution | Description |
| 325 | Low cost no electrical/no fuel consuming refrigerators to store food at home level. | 61.90 | 0.53 | Other Innovations | Development |
| 326 | What is the potential for developing affordable, effective rapid diagnostic technologies to test for diarrheal pathogens? | 61.89 | 0.55 | Other Innovations | Description |
| 327 | Evaluation of alternate ORS formulations containing complex carbohydrate and peptide/protein in ORS. | 61.85 | 0.59 | Case Management | Development |
| 328 | Potential for development of diagnostic to identify children with diarrhoea who would likely benefit from antibiotics? (this is different from aetiology; this is to explore whether a subset of children with diarrhoea can be identified in whom antibiotics might prevent a more adverse outcome) | 61.85 | 0.55 | Diagnostics | Description |
| 329 | Strategic regional protocols for the measurement of antibiotic resistance in different geographic regions with distribution of results locally and at centralized sites. | 61.80 | 0.66 | Disease Burden, Aetiology & Distribution | Delivery |
| 330 | What is the role of intestinal microbiome in regulating bacterial translocation/inflammation/immunity of the small intestine following enteric infections and undernutrition? | 61.77 | 0.47 | Nutrition & Long-Term Outcomes | Discovery |
| 331 | Develop improved markers for the measures of epithelial damage/turnover to measure the physiological insult associated with different enteropathogens. | 61.71 | 0.60 | Disease Burden, Aetiology & Distribution | Discovery |
| 332 | How to assess the potential of host immune modulators for anti-diarrhoeal therapy? | 61.71 | 0.44 | Emerging Interventions | Discovery |
| 333 | What products and gadgets can facilitate effective hand cleansing? | 61.46 | 0.48 | WASH Interventions | Development |
| 334 | While the GEMS program provides impressive documentation on the aetiology of diarrheal morbidity and mortality in developing countries among children less than 5 years of age which will serve as a basis for decision making on vaccine development priorities, there is a clear gap of knowledge regarding the host immune response to the various antigens in frame of single and mixed natural infections in the same settings. Since most, if not all of the enteric natural infections induce protective immunity for a limited time, under a proper epidemiological design (e.g. cases versus health and exposed controls), correlates or surrogates of protection can be developed in these specific populations and further used for vaccine development evaluation. Should resources be channelled in this direction? | 61.40 | 0.59 | Vaccines | Development |
| 335 | Conduct trials to evaluate the efficacy of pre- and pro-biotics for the clinical outcome of non-antibiotic associated diarrhoea. | 61.12 | 0.59 | Case Management | Development |
| 336 | What are the modes of transmission of multi-resistant strains of Shigella? | 61.10 | 0.58 | Disease Burden, Aetiology & Distribution | Description |
| 337 | Does infection with multiple enteric pathogens contribute to diarrhoea pathogenicity? | 61.07 | 0.56 | Disease Burden, Aetiology & Distribution | Description |
| 338 | In view of the diminished immunogenicity of oral enteric vaccines in children in developing countries, should significant resources be allocated to find out if non-oral stimulation of intestinal immune response will also be diminished in these populations as compared to young children in developed countries? | 61.06 | 0.56 | Vaccines | Development |
| 339 | Are there easily recognizable signs/symptoms of an acute diarrhoea episode that predict which episodes will progress to dehydration or severity in need of IV treatment and/or hospitalization? | 61.03 | 0.52 | Case Management | Description |
| 340 | Given the difficulty in effectively immunizing infants and young children in developing countries with oral vaccines, can the skin (transcutaneous or intradermal immunization), sublingual or more classic IM route of delivery get around environmental enteropathy issues and improve vaccine “take” and effectiveness? | 60.84 | 0.55 | Vaccines | Development |
| 341 | There are other potential etiologies of the depressed immune response to live oral vaccine that could be explored. The so-called “environmental enteropathy” (EE) is a highly prevalent condition among children in developing countries characterized by small bowel histopathology, proximal small intestinal bacterial overgrowth, intestinal malabsorption and possible increased intestinal permeability. EE is associated with nutritional deficiencies, growth stunting, and most probably a dysregulation of the immune responses/tolerance balance. Altered intestinal microbiota is one factor associated with EE that might play a role in the ability to mount a mucosal immune response, as described in #2 above. Are there merits to defining the most important elements of EE that are associated with the inability to mount a vigorous immune response to oral vaccines, such as biomarkers of gut inflammation (as measured by the lactulose/rhamnose [L/R] 6 hour urine test, α1 anti-trypsin, serum zonulin, micronutrient deficiencies and inflammatory markers such as C-reactive protein [CRP])? | 60.84 | 0.53 | Vaccines | Discovery |
| 342 | What is the aetiology of the ~40% of cases of diarrhoea that is still of unknown aetiology? | 60.79 | 0.61 | Emerging Interventions | Discovery |
| 343 | Is there a role for non-antibiotic therapy (bismuth, probiotics, etc.) in preventing long-term complications of early childhood diarrhoea? | 60.79 | 0.49 | Diagnostics | Development |
| 344 | Solar refrigerators to prevent food contamination. | 60.70 | 0.47 | Other Innovations | Discovery |
| 345 | How can countries best develop their own policy on domestic water supply requirements? | 60.63 | 0.53 | WASH Interventions | Delivery |
| 346 | Efficacy of using demonstration videos during counselling to improve responsible feeding for prevention of childhood diarrhoeal disease. Assess the relationship and impact of improving diet quality on diarrhoeal burden. | 60.63 | 0.49 | Preventive Nutrition Strategy | Development |
| 347 | Direct mucosal stimulation at present is the most affordable and direct way to develop mucosal responses but suffers from a number of shortcomings that may include lower and shorter efficacy.  Impediments to vaccine “take”, processing and response may be intrinsically affected by the intestinal alterations associated with environmental enteropathy (altered absorption/permeability and inflammation).  What are the mechanisms of this effect and strategies to overcome these effects at the mucosal surface? Are there strategies to overcome these effects through combination or alternative immunization approaches? | 60.60 | 0.51 | Vaccines | Discovery |
| 348 | Is zinc for the treatment of diarrhoea also effective in populations without zinc deficiency? | 60.43 | 0.56 | Case Management | Development |
| 349 | How much intestinal barrier breakdown and intestinal bacterial translocation could influence brain development in undernourished (immunocompromised) and chronically infected children? Is there any independent effect of secretory, dehydrating diarrhoea regarding undernutrition in growing children? | 60.40 | 0.46 | Nutrition & Long-Term Outcomes | Discovery |
| 350 | Remote health centres have been encouraged to give ORS via naso-gastric tubes prior/during transfer to district hospitals. Could other techniques be developed to give fluids used more effectively with readily available items in remote settings? | 60.38 | 0.49 | Other Innovations | Delivery |
| 351 | Characterize invasive/non-invasive disease as it affects cognition. | 60.36 | 0.59 | Nutrition & Long-Term Outcomes | Description |
| 352 | What is the effect of maternal depression on episodes of diarrhoeal disease? | 60.35 | 0.55 | Nutrition & Long-Term Outcomes | Description |
| 353 | Establish the role of probiotics in intestinal flora development and diarrhoea, what is their role in diarrhoeal prevention? | 60.34 | 0.55 | Preventive Nutrition Strategies | Development |
| 354 | Combine all WASH activities to develop integrated campaigns using cross-sectoral partners (NGOs, public and private sectors). | 61.22 | 0.57 | Other Innovations | Development |
| 355 | How to maximize opportunities resulting from over-the-counter (OTC) classification of zinc treatment? | 60.15 | 0.41 | Monitoring & Evaluation | Delivery |
| 356 | What is the balance of inflammation/innate immunity/parasite killing and intestinal recovery? | 59.70 | 0.43 | Nutrition & Long-Term Outcomes | Discovery |
| 357 | What are the best clinical study designs and analytical methods to determine the diarrhoeal aetiology in developing countries: case/control with odds ratios, quantitative diagnostics, prospective studies? | 59.69 | 0.50 | Diagnostics | Description |
| 358 | What is the role of quantitative diagnostics in inferring diarrhoeal aetiology in the setting of mixed infections? | 59.58 | 0.52 | Diagnostics | Development |
| 359 | More affordable and nutritious infant/young child feeding products. | 59.53 | 0.46 | Other Innovations | Discovery |
| 360 | Despite the lower efficacy in randomized controlled field trials in sub-Saharan Africa and South and Southeast Asia as compared with similar trials in developed countries, it is expected that oral rotavirus vaccines will have a significant impact on diminishing disease burden in these regions. However, it would be of much importance to assess potential rotavirus vaccination-driven changes in the attributable fraction of the various enteropathogens in the burden of diarrheal diseases morbidity and mortality in the GEMS countries with existent pre-vaccination era baseline data. Particular attention should be given to "replacement" of rotavirus genotypes included in the vaccine used and the potential emergence of noroviruses (or other enteric viruses). Should resources be channeled in these directions? | 59.40 | 0.56 | Vaccines | Description |
| 361 | What impact does the gut microbiome have on susceptibility to severe diarrhoea in the infant? | 59.31 | 0.55 | Disease Burden, Aetiology & Distribution | Discovery |
| 362 | What is the physiopathology/mediators of infected brain/small intestine axis vicious cycle leading to poor growth and cognitive decline in children? | 59.24 | 0.44 | Nutrition & Long-Term Outcomes | Discovery |
| 363 | What is the consequence of intestinal permeability increase in diarrhoeal disease in a) children with wasting b) children with stunting c) well-nourished children? | 59.14 | 0.57 | Disease Burden, Aetiology & Distribution | Discovery |
| 364 | How best to evaluate the impact of the use of anti-emetics in childhood enteritis? | 59.10 | 0.49 | Emerging Interventions | Development |
| 365 | Micro-finance for water filters/treatment products (including chlorine) and sanitation. Offer a range of products using NGOs, self-help groups, micro-finance groups and the private sector to promote. | 58.97 | 0.45 | Other Innovations | Delivery |
| 366 | Are there differences in diarrhoea aetiology by antibiotic use rates (i.e. has antibiotic resistance changed the pathogen profile)? | 58.90 | 0.57 | Disease Burden, Aetiology & Distribution | Description |
| 367 | Using advanced methodologies (e.g. combining metanolomics, proteomics and transcriptomics) can we better understand the relationship between nutrition and immunity to acute enteric infections? | 58.82 | 0.49 | Emerging Interventions | Discovery |
| 368 | What clinical trials of dioctahedral smectite (natural hydrated aluminomagnesium silicate) should be started to evaluate the impact on need for intravenous fluids, hospitalization and costs? | 58.67 | 0.51 | Emerging Interventions | Development |
| 369 | With the vast increase in sensitivity (and demonstration of co-infections) found with PCR-based technologies, how do we defined “clinically relevant,” and “pathogen?” Can quantitative cut-offs be set for clinical relevance? Will these need to be age-based? | 58.63 | 0.51 | Diagnostics | Description |
| 370 | What animal husbandry practices are most associated with the transference of antibiotic resistance to important human pathogens? | 58.62 | 0.56 | Disease Burden, Aetiology & Distribution | Description |
| 371 | Why has effective treatment of children with diarrhoea stagnated (and is some cases declined) in many countries? | 58.58 | 0.56 | Case Management | Description |
| 372 | Public-private partnerships for promoting ORS and zinc treatment. Public sector and donors take risk for developing a new market. | 58.49 | 0.48 | Other Innovations | Delivery |
| 373 | Association of exposure to environmental toxicants (As, Cd) with chronic diarrhoea and its effect on later cognition and school performance. | 58.48 | 0.57 | Nutrition & Long-Term Outcomes | Description |
| 374 | How can we quantify the impact of water scarcity on public health? | 58.46 | 0.49 | WASH Interventions | Description |
| 375 | Are there standard indicators that can measure progress in both public and private sector programs across both facility-based and community-based programs, in order to facilitate comparability across interventions? | 58.44 | 0.42 | Monitoring & Evaluation | Description |
| 376 | Can oral immunization practices be improved to enhance vaccine take through more effective buffering with less volume and greater temperature stability? Does mucosal adjuvant development and testing warrant more funding support? | 58.35 | 0.53 | Vaccines | Development |
| 377 | How should practical ways to promote the secretion of antibacterial peptides and a new therapy be developed? | 58.18 | 0.44 | Emerging Interventions | Development |
| 378 | Cell mediated immunity (CMI) parameters including T and B cell memory should be measured following natural and vaccine induced immune response towards identification of single or combined correlates of protection against diarrhoeal diseases. Relatively increased volumes of whole blood are needed for these measurements, making them difficult to perform in paediatric populations. Should resources be allocated to develop assays, which will work on minimal amounts of blood, either for direct quantification of cytokines or for indirect assessment of their production (mRNA). The same for large scale use of functional assays? | 58.16 | 0.55 | Vaccines | Discovery |
| 379 | Develop interventions that will reduce bacterial contamination of crops irrigated with contaminated water in developing countries. | 58.10 | 0.52 | Other Innovations | Discovery |
| 380 | The diarrheal disease burden and aetiology are not well known in most of the countries in sub-Saharan Africa. In addition, except in some special situations such epidemics of cholera or bloody diarrhea, diarrheal diseases, in general are not considered as part of a national routine surveillance system, therefore, have less change of not being discussed on a regular basis during the national decision-making meetings. Pathogens like *Shigella, Cryptosporidium,* diarrheagenic *Escherichia coli, Camplylobacter jejuni* and gastroenteritis viruses are not known by the health progessionals and this has an impact on the diagnosis and case management of diarrhoea in the field. What could be done to improve this situation? | 57.95 | 0.26 | Vaccines | Description |
| 381 | Are enteroaggreative E. coli (EAEC) and classical and non-classifical enteropathogenic E. coli (EPEC) causes of diarrhoea? | 57.80 | 0.94 | Disease Burden, Aetiology & Distribution | Description |
| 382 | Determine the mechanisms of multi-resistance in strains of Shigella. | 57.72 | 0.66 | Disease Burden, Aetiology & Distribution | Discovery |
| 383 | Are there significant, currently unrecognized pathogens in no-pathogen identified diarrhoeal stools? If so, how can these pathogens be more readily detected? | 57.55 | 0.50 | Diagnostics | Discovery |
| 384 | Since we need better immunological tools to assess and predict vaccine efficacy in the field, should more investment go towards the development of high-throughput functional assays for assessing enteric vaccine immunogenicity? | 57.34 | 0.58 | Vaccines | Development |
| 385 | How can we ensure sufficient water quantity for WASH in the context of expanding populations and climate change? | 57.31 | 0.48 | WASH Interventions | Development |
| 386 | Research to identify and prevent nutritional and other risk factors for poor immune response to rotavirus vaccines. | 57.25 | 0.43 | Preventive Nutrition Strategies | Description |
| 387 | Would markers of intestinal inflammation be useful as diagnostic indicators for the identification of severe childhood diarrhoea? If so, are there markers that might be useful which would allow physicians to monitor disease severity in breast-fed as well as non-breast-fed infants? | 57.14 | 0.51 | Diagnostics | Development |
| 388 | What factors have contributed to the reduction in Shigella disease burden globally over the last two decades? | 57.07 | 0.58 | Disease Burden, Aetiology & Distribution | Description |
| 389 | Develop a food, better than the usual fortified blended foods, for food insecure populations to reduce the incidence of diarrhoeal disease. | 56.97 | 0.48 | Preventive Nutrition Strategies | Development |
| 390 | Children from developing countries have been shown to exhibit depressed immune responses to live oral vaccines, including vaccines against cholera, polia, *Shigella,* and rotavirus. Several hypotheses have been proposed to account for this phenomenon, including co-infection with intestinal helminths, interference from antibody and other antimicrobials in breast milk, and proximal small intestinal bacterial overgrowth. The relationship between the intestinal microbiota and a host of inflammatory and auto-immune conditions is an active area of research, with the hopes that better understanding of this process can lead to interventions aimed to restore a healthy intestinal microbiota. Available data suggest that the intestinal microbiota interact with the innate and regulatory cell differentiation in the gut that is critically involved in maintaining immune tolerance. Accordingly, one might ask “is the composition of intestinal microbiota in children from developing countries an important determinant of the ability to mount a vigorous immune response to oral vaccination?” | 56.89 | 0.54 | Vaccines | Discovery |
| 391 | Can acute diarrhoeal diseases be managed by altering the gut microbiota? (What organisms predominate in the guts of individuals with acute diarrhoeal disease? Can an imbalanced microbiota be restored to normal by supplementing with either appropriate organisms through fecal transplants?) | 56.87 | 0.48 | Emerging Interventions | Development |
| 392 | Clinical trials to evaluate the efficacy of zinc in the treatment of diarrhoea due the different pathogens, i.e. rotavirus, cholera, etc. | 56.75 | 0.58 | Case Management | Development |
| 393 | How can self supply and household water treatment be scaled up effectively? | 56.68 | 0.42 | WASH Interventions | Development |
| 394 | What are the effects of repeated infection (symptomatic or asymptomatic) on the gut mucosal immune response (either to vaccines or to natural infection) and on innate immune response and on systemic immune response? | 56.42 | 0.59 | Disease Burden, Aetiology & Distribution | Discovery |
| 395 | What could be the most effective vaccine candidates against diarrhoeal pathogens to be delivered through the EPI in Africa and Asia? Parenteral conjugates? Mucosal vaccines? Combination of vaccines? | 56.42 | 0.45 | Vaccines | Discovery |
| 396 | To what extent have antibiotic resistant enterics such as ESBL E. coli and CRE and C. difficile become a problem in children in developing countries? | 56.40 | 0.55 | Diagnostics | Description |
| 397 | Cost-effectiveness of surveillance for pathogens that may result in epidemics of diarrhoea. | 56.17 | 0.65 | Disease Burden, Aetiology & Distribution | Description |
| 398 | What are pathogenic mechanisms of diarrhoea caused by viruses other than rotavirus? | 56.15 | 0.56 | Disease Burden, Aetiology & Distribution | Discovery |
| 399 | In the view of the revelation of the apparent importance of *Cryptosporidium* as a major etiologic agent of paediatric diarrhoea in developing countries, should major investments be made to study human host-parasite interactions and immunity to guide *Cryptosporidium* vaccine developments? | 56.00 | 0.58 | Vaccines | Description |
| 400 | What is the role of the gut microbiome in identifying children at higher risk of diarrhoea and is this role separate from breastmilk-induced changes? | 55.60 | 0.46 | Preventive Nutrition Strategies | Discovery |
| 401 | What is the role of arginase in pathways to improve the intestinal barrier structure and function? | 55.60 | 0.46 | Nutrition & Long-Term Outcomes | Discovery |
| 402 | Redefine “disease” as impaired growth trajectory, rather than just as “diarrhoea” (overt ‘squirts’) and reassess etiologies, epidemiology and impact with this new “case definition.” | 55.47 | 0.46 | Nutrition & Long-Term Outcomes | Desrciption |
| 403 | What is the impact of high or low-grade inflammation of proinflammatory or repair genetic markets (such as that seen with Shigella or Cryptosporidium infections, respectively) with or without diarrhoea on growth trajectories and/or cognitive development in children in impoverished endemic areas? | 55.43 | 0.43 | Nutrition & Long-Term Outcomes | Discovery |
| 404 | Recent findings from the GEMS demonstrate that the highest diarrhoea-related mortality occurs in regions with the highest HIV prevalence. Is it realistic to assume that an increasing proportion of children ill receive antiretroviral therapy in the near future? If so, should we invest resources in trying to better understand the contribution of untreated HIV infection to diarrhoeal morbidity and mortality in order to better prepare for the epidemiology of diarrhoea that is expected to exist in developing countries 10 years from now, when HIV has become a chronic, treated condition (and rotavirus is widely implemented)? | 55.36 | 0.47 | Vaccines | Discovery |
| 405 | What metabolic profiles of gut microbiome are related to improved childhood growth and/or decreased diarrhoeal incidence? | 55.34 | 0.58 | Disease Burden, Aetiology & Distribution | Discovery |
| 406 | Much of what is known about the epidemiology of diarrhoea in developing countries is derived from studies conducted in centers with well-established research programs and a strong microbiology infrastructure. To better understand whether the results of these studies are generalizable to more remote areas of the developing world where the majority of children leave, is it feasible and compelling to design studies to measure the burden, aetiology and sequelae of diarrhoeal disease among children under 5 years of age living in remote regions of developing countries, using a molecular toolkit of diagnostics applied to the field? | 55.24 | 0.51 | Vaccines | Description |
| 407 | Conduct studies on the impact of policy and the role of policy change in the delivery of preventive zinc supplementation. | 55.22 | 0.52 | Preventive Nutrition Strategies | Delivery |
| 408 | How will climate change affect WASH at international, national and local levels and what will be the effect on diarrhoeal disease? | 55.12 | 0.51 | WASH Interventions | Description |
| 409 | Identification of different components of breast milk (lactoferin, lysosome, oligosaccharides) with anti-inflammatory/infective properties in relation to their effect on diarrhoeal disease. | 55.02 | 0.46 | Preventive Nutrition Strategies | Discovery |
| 410 | In treating diarrhoea, when should anti-peristalsis drugs be employed and for how long? | 55.01 | 0.51 | Emerging Interventions | Development |
| 411 | Are more field-friendly diagnostics for assessing disease burden of enteric pathogens needed? After rotavirus, many developing countries do not have a very clear sense of how other enteric pathogens like ETEC, *Shigella* and *Cryptosporidium* impact on infant and child health. This information is important in the development of potential markets for utilization of these vaccines as they become available and also to further justify their development. | 54.95 | 0.52 | Vaccines | Description |
| 412 | Explore outcome-based disincentives to foster a no-tolerance policy among communities for diarrhoeal deaths:   - Community campaigns urging to report all diarrhoea-related deaths to central committee (e.g. panchayat) - System of fines for providers associated with higher rates of death among treated patients - Rewards/recognition for providers associated with lower death rate | 54.84 | 0.41 | Other Innovations | Delivery |
| 413 | Subunit vaccines against enteric or other pathogens arguably present one of the most well defined approaches to vaccine development. Certainly, the emergence of so-called ‘reverse vaccinology’ approaches are placing many such candidates into the vaccine development pipeline. Would a concerted effort to develop potent, safe mucosal vaccines that are suitable or perhaps optimized for use in paediatric populations alter the likelihood of success with such subunit vaccines? | 54.23 | 0.50 | Vaccines | Discovery |
| 414 | What are the ways in which private sector business and marketing can contribute to sanitation and hygiene improvements? | 54.23 | 0.42 | WASH Interventions | Delivery |
| 415 | Given the limitations in health expenditures in less developed countries where the burden of enteric diseases in greatest, even the availability of safe, efficacious, relatively inexpensive individual vaccines against the most common enteric infectious causes of childhood morbidity and mortality may present intractable cost and logistical issues for implementation. Are there merits to earlier investment in platform or combination vaccine technologies to foster multi-pathogen vaccine development vs. simply delaying such efforts until one or more pathogen-specific vaccines become available? | 54.16 | 0.50 | Vaccines | Description |
| 416 | What features of the enteric microbiome are related to intestinal function and physical growth? | 54.07 | 0.56 | Disease Burden, Aetiology & Distribution | Discovery |
| 417 | How has external funding for program changes impacted coverage of ORS for diarrhoea over time? | 54.05 | 0.49 | Case Management | Description |
| 418 | What are genetic markers of predisposition to worsened diarrhoea an can these be used to identify more effective interventions? | 53.95 | 0.43 | Nutrition & Long-Term Outcomes | Discovery |
| 419 | Develop low cost measures to assess intestinal mucosal integrity and risks for prolonged/persistent diarrhoea. | 53.86 | 0.45 | Diagnostics | Description |
| 420 | How much do maternal stress and depressive symptoms influence child nutrition and diarrhoea? | 53.53 | 0.48 | Preventive Nutrition Strategies | Description |
| 421 | Are private pharmaceutical laboratories (companies) able to effectively market ORS and zinc or is it more effective for them to serve as contract manufacturers for a social marketing company? | 53.28 | 0.52 | Monitoring & Evaluation | Delivery |
| 422 | How to assure human capacity for WASH at every level? | 53.23 | 0.46 | WASH Interventions | Development |
| 423 | For diarrhoeal disease pathogens such as *Shigella,* enterotoxinogenic *Escherichia coli* and *Campylobacter jejuni* that are notorious for their antigenic heterogeneity, should common protein antigen strategies be pursued as a high priority in vaccines development even though Mother Nature does not appear to confer protection via immune responses to these common antigens? Should we be optimistic that we can do better than Mother Nature (as in the case with tetanus toxoid)? | 53.17 | 0.56 | Vaccines | Discovery |
| 424 | Cyclical (every 2 years) propagated extensive *Shigella sonnei* shigellosis epidemics have occurred in Israel in the last 15 years, replacing previously occurring common source or combined common source-propagated epidemics. Children 1-4 years of age living in poor and crowded conditions and mostly affected. Should resources be invested on household and kindergarten-based epidemiological studies to identify immunological, gut microbiota and genetic associated correlates of protection in immunocompetent and fairly nourished children as a reference model? | 52.67 | 0.52 | Vaccines | Description |
| 425 | Combination of enteric licensed or investigational vaccines given by more than one route (e.g. parenterally and orally), a form of heterologous prime-boost, may improve immunogenicity and protective efficacy in children in developing countries (one classical example is the occasional use of IPV and OPV dosing against polio). Usually this approach is not enthusiastically embraced by vaccine manufacturers and is a logistical management challenge for the EPI in developing countries. Should research resources be channelled in this direction? | 52.25 | 0.48 | Vaccines | Development |
| 426 | Education/behaviour modification research to increase utilization of refrigerators for storage of weaning foods. | 52.22 | 0.51 | Other Innovations | Development |
| 427 | In order to be able to convince developing country governments to consider implementing new vaccines against diarrhoeal infections, criteria such as immunogenicity, composition (presence of pork product in some vaccines would be problematic in some countries), mode of administration (oral vs. parenteral injection), presentation, need for cold chain, number of doses required, duration of immunity conferred, potential combination with other vaccines in the same syringe and concomitant administration with routine EPI vaccines, must be considered today. What is the best way to prioritize which of these parameters comprise the most critical for vaccines intended to prevent diarrhoeal disease in developing country children? | 51.79 | 0.43 | Vaccines | Delivery |
| 428 | Is there consensus on the best approaches for evaluation of new molecular and broad-based technologies? | 51.73 | 0.46 | Diagnostics | Discovery |
| 429 | Identify nutrients besides zinc that aid in the prevention, or alter the duration, of diarrhoea. | 51.72 | 0.43 | Preventive Nutrition Strategies | Discovery |
| 430 | Cell phones, portable DVD players and other interventions to access information. Use technology to improve information flow to caregivers. | 51.64 | 0.47 | Other Innovations | Development |
| 431 | Find practical ways to foster secretion of antibacterial peptides as a new therapy. | 51.63 | 0.45 | Case Management | Discovery |
| 432 | What are the best approaches for monitoring emerging pathogens in the gastrointestinal tract and who should do this? | 52.60 | 0.50 | Diagnostics | Description |
| 433 | Which drugs should be developed to activate δ-opiate receptors, thereby decreasing secretion without slowing intestinal transfer or promoting overgrowth? | 51.50 | 0.41 | Emerging Intervention | Discovery |
| 434 | Does the epigenetic interaction between enteric pathogens and infant protect or predispose children to severe diarrhoea? | 51.25 | 0.64 | Disease Burden, Aetiology & Distribution | Discovery |
| 435 | What are immunoendocrine pathways through which enteric infections (symptomatic and asymptomatic) modulate linear growth? | 50.54 | 0.58 | Disease Burden, Aetiology & Distribution | Discovery |
| 436 | What genes/signalling pathways are involved in poor intestinal responses/improved mucosal recovery after enteric infections? | 50.18 | 0.44 | Nutrition & Long-Term Outcomes | Discovery |
| 437 | What is the normal enteric virome in both the developing and developed world? | 49.70 | 0.51 | Emerging Interventions | Discovery |
| 438 | What is the potential for zinc in the treatment of adult colds – as a strategy to increase zinc volumes globally and incentivize manufacturers to increase their investment in zinc sales/promotion overall? | 49.30 | 0.45 | Other Innovations | Delivery |
| 439 | Complete reclassification of Blastocystis hominis to provide basis of test to separate and identify pathogenic strains or species present. | 48.69 | 0.49 | Diagnostics | Description |
| 440 | What are the genetic markers of predisposition to worsened diarrhoea and can these be used to identify clinical features of increased risk? | 48.06 | 0.49 | Nutrition & Long-Term Outcomes | Description |
| 441 | Study the effect of probiotics on the mucosal and systemic immune response. | 48.00 | 0.54 | Case Management | Discovery |
| 442 | Recent findings from the GEMS case-control study of moderate-severe diarrhoea (MSD) show a consistent association between *Giardia* infection, as detected by ELISA, and control status, suggesting that *Giardia* may play a role in protecting children from diarrhoeal disease. Is there a safe and ethical methodology to pursue the hypothesis that chronic *Giardia* infection during childhood protects children from infectious diarrhoea? Conversely, could one postulate that *Giardia* infection inhibits colonization and immune responses following oral inoculation with live vaccines? | 47.82 | 0.48 | Vaccines | Development |
| 443 | How have messages on avoidance of mixed feeding in infants with HIV influenced messages for prevention/management of dehydration? | 47.47 | 0.46 | Preventive Nutrition Strategies | Development |
| 444 | Virulence and pathogenicity of Aeromonas isolated from environmental and diarrhoeal cases. | 47.44 | 0.64 | Disease Burden, Aetiology & Distribution | Description |
| 445 | What is the role of Aeromonas in diarrhoea? | 46.98 | 0.91 | Disease Burden, Aetiology & Distribution | Description |
| 446 | For young children in developing countries, a number of enteric agents can cause early, repeated and sometimes persistent infection, contributing to altered intestinal form, function and physiology. This in turn may lead to increased susceptibility to enteric infection and illness. If prevention of rotavirus, perhaps in addition to the next one or two most prevalent enteropathogens in early life, has a significant salutary effect on gut health, nutritional status and non-specific resistance to intestinal infections, it would be import to know. Using rotavirus vaccination as a paradigm, can this question be studied in a way that informs priorities in vaccine development? | 46.96 | 0.48 | Vaccines | Description |
| 447 | Determine the mechanism by which some pathogens like *Shigella dysenteraie* type 1 or *Vibrio cholera* O139 fade out and for how long. | 46.87 | 0.89 | Disease Burden, Aetiology & Distribution | Description |
| 448 | Develop guidelines for testing asymptomatic children for potential pathogen carriage and follow up – opportunities for implementation will vary depending on geographic area and resources. An example would be a rapid test for the microsporidia (Enterocytozoon bienuesi, Encephalitozoon intestinalis). | 46.87 | 0.49 | Diagnostics | Development |
| 449 | Can GPS be used to identify higher incidence sites of diarrhoea? | 46.85 | 0.47 | Other Innovations | Description |
| 450 | How to count the investment of the private sector in WASH? | 45.92 | 0.46 | WASH Interventions | Description |
| 451 | Ultimately, major impacts of successful enteric vaccines would translate into reduced mortality and reduced acute and chronic morbidity (e.g., DALYs). Adding to the general consternation, early effective treatment of infectious diarrhoea should preclude most acute deaths. Hwo do we develop improved, ethically sound, methods for measuring mortality reduction in Phase 3 and 4 clinical trials of new enteric vaccines. | 45.49 | 0.42 | Vaccines | Description |
| 452 | Based on recent findings from the “GEMS” and MAL-ED” studies that show an unexpectedly large burden of paediatric diarrhoea due to *Cryptosporidium* in developing countries, should resources be channelled into a crash, accelerated program for *Cryptosporidium* vaccine development? | 44.86 | 0.49 | Vaccines | Description |
| 453 | Which means should be supported in the development of new antiviral drugs? | 44.71 | 0.41 | Emerging Interventions | Discovery |
| 454 | Improve value chain for waste collection, transport, disposal, re-use. | 44.48 | 0.69 | Other Innovations | Development |
| 455 | Identify and define the roles of anti-inflammatory agents in the management of shigellosis and other invasive diarrhoeas. | 43.87 | 0.61 | Other Innovations | Discovery |
| 456 | What are the non-infectious causes of diarrhoea in developing countries (incidence/prevalence/severity)? | 43.79 | 0.58 | Disease Burden, Aetiology & Distribution | Description |
| 457 | What is the role for surveillance augmentation, especially for vaccine resistant mutants? | 43.78 | 0.55 | Emerging Interventions | Description |
| 458 | Should diarrhoea mortality in age groups older than 5 years be considered as a secondary factor in targeting enteric vaccine development efforts? | 43.57 | 0.45 | Vaccines | Description |
| 459 | What information from the Human Microbiome project could be used to develop diagnostic tests or therapeutic regimens? | 42.04 | 0.43 | Diagnostics | Discovery |
| 460 | Another perspective on oral live vaccines is offered as a question to be answered. The constraint (diminished immunogenicity and effectiveness) may relate to poor infectiousness on gut microbiota rather than to immune responses after gut mucosal infection. That may indeed be a heightened innate immunity due to repeated infections, etc. So, can improved live oral vaccines for children in developing countries be achieved by aiming to increase the infectiousness of the attenuated agents while not removing all the virulent counterparts? | 41.09 | 0.43 | Vaccines | Development |
| 461 | What impact does breast milk fortification with novel anti-inflammatory agents have on diarrhoeal prevention and outcome? | 41.74 | 0.40 | Preventive Nutrition Strategies | Discovery |
| 462 | Are there significant, currently unrecognized or unappreciated diarrhoea-causing enterotoxins in food stuffs or environmental materials consumed by children? If so, how can these toxins be detected? | 40.77 | 0.45 | Diagnostics | Discovery |
| 463 | Significant intestinal adaptions and wasting can occur within the first 6 months of life among infants in resource-limited settings. It is thought that this is due to chronic and/or repetitive exposure to poorly understood stimuli. Can these stimuli be identified? Can a vaccine be developed that doesn’t target specific pathogens but that prevents the effects of these stimuli (i.e., an anti-environmental enteropathy vaccine)? Such an approach could facilitate the ability to generate subsequent vaccine “takes” and could have many additional beneficial sequelae. | 40.00 | 0.41 | Vaccines | Discovery |
| 464 | In a popular television serial/soap opera, create a storyline with adversarial relationship between mother-in-law and daughter-in-law. Dramatized story of conflict over child with diarrhoea when daughter-in-law saves the day by giving correct treatment to child (after mother-in-law’s advice leads to near death of child), winning admiration of husband and family. | 39.59 | 0.45 | Other Innovations | Delivery |
| 465 | What is the role of organ dysfunction (liver, pancreas) among children with high diarrhoeal burdens and malnutrition? | 39.09 | 0.49 | Preventive Nutrition Strategies | Discovery |
| 466 | What are the genetic factors that influence recovery from childhood diarrhoea? Susceptibility? Mortality? | 36.96 | 0.55 | Diagnostics | Description |

**Supplementary Table S16 – Team Composition, Including Team Leaders, Participants, Countries Represented and Institutional Affiliations**

| Area of focus: | Team Leader(s) and Institutional Affiliations | Global Expert Participants |
| --- | --- | --- |
| Disease Burden, Aetiology and Distribution | Robert Black  Edgar Berman Professor and Chair of the Department of International Health and Director of the Institute for International Programs of the Johns Hopkins Bloomberg School of Public Health | - Anuradha Bose, Christian Medical College, India - Sandy Cairncross, London School of Hygiene and Tropical Medicine, UK - Laura Caulfield, Johns Hopkins University, USA - Christa Fischer-Walker, Johns Hopkins University, USA - Birger Forsberg, Karolinska Institute, Sweden - Richard Guerrant, University of Virginia, USA - Sharon Hutley, London School of Hygiene and Tropical Medicine, UK - Gagandeep Kang, The Wellcome Research Laboratory, India - Margaret Kosek, Johns Hopkins School of Public Health, USA - Claudio Lanata, Nutritional Research Institute, Peru - Dennis Lang, Fogarty International Center, USA - Steve Luby, ICDDRB, Bangladesh - Umesh Parashar, Centre for Disease Control, USA - Stefan Peterson, Karolinska Institute, Sweden - Zeba Rasmussen, Fogarty International Center, USA - Alexander Rowe, National Center for Infectious Diseases, USA - Halvor Sommerfelt, Center for International Health, Norway - Mahesh Srinivas, Micronutrient Initiative, Canada |
| Nutrition and Long-Term Outcomes (DALYs) | Richard Guerrant  Founder and Director of the Center for Global Health at the University of Virginia School of Medicine, Thomas H. Hunter Professor of International Medicine in the Division of Infectious Diseases and International Health | - Mark DeBoer, University of Virginia, USA - Richard Guerrant, University of Virginia, USA - Beena Koshy, Christian Medical College, India - Jonathan Mitchell, Concentric Development, USA - Sean Moore, Cincinnati Children’s Medical Center, USA - Reinaldo Oria, Federal University of Ceara, Brazil - Zeba Rasmussen, Fogarty International Center, USA - Aisha Yousafzai, Aga Khan University, Pakistan - Rebecca Scharf, University of Virginia, USA - Fahmida Tofail, ICDDRB, Bangladesh |
| Preventive Nutrition Strategies (Including Zinc) | Zulfiqar Bhutta  The Husein Laljee Dewraj Professor and Founding Chair, Division of Women and Child Health, The Aga Khan University, Visiting Professor at the Programme for Global Paediatric Research, SickKids International | - Jay Berkley, KEMRI-Wellcome Trust, Kenya - Robert Bandsma, University Medical Centre Gronigan, Netherlands - Tahmeed Ahmed, ICDDRB, Bangladesh - Cholpon Imanalieva, UNICEF, Kyrgyzstan - Ferdinand Haschke, Nestle, Switzerland - Rukhsana Haider, ICDDRB, Bangladesh - Mike English, KEMRI-Wellcome Trust, Kenya - Etienne Nel, Stellenbosch University, South Africa - Durre Samin Akram, Health Education and Literacy Program, Pakistan - David Brewster, Canberra Hospital, Australia - Chris Duggan, Harvard University, USA - Kim Harding, Micronutrient Initiative, Canada - Angela Okolo, University of Benin, Nigeria - Emorn Wasantwisut, Mahidol University, Thailand - Dipika Sur, National Institute of Cholera and Medical Diseases, India |
| Diagnostics | William Petri  Wade Hampton Frost Professor of Medicine, Chief of the Division of Infectious Diseases and International Health at University of Virginia, President of the American Society of Tropical Medicine and Hygiene. | - Peter Gilligan, University of North Carolina Medical Center, USA - Asad Ali, Aga Khan University, Pakistan - Karen Carroll, Johns Hopkins University, USA - Lynne Garcia, UCLA Medical Center, USA - Shinjiro Hamano, University of Nagasaki, Japan - Yiwu He, Gates Foundation, USA - Beth Kirkpatrick, University of Vermont, USA - David Lyerly, Techlab Inc., USA - Carl Mason, Armed Forces Research Institute of Medical Sciences, Thailand - Alexander McAdam, Harvard University, USA - Stefan Peterson, Karolinska Institute, Sweden - Megan Reller, Johns Hopkins University, USA - Theodore Steiner, University of British Columbia, Canada - Mehmet Tanyuksel, Gulhane Military Medical Academy, Turkey - JJ Verweij, Leiden University, Netherlands - Eric Houpt, University of Virginia, USA |
| Vaccines for Diarrhoea Prevention | Myron Levine  Director of the Center for Vaccine Development, Head of Geographic Medicine, University of Maryland | - George Armah, Noguchi Memorial Institute for Medical Research, Ghana - Louis Bourgeois, PATH, USA - Marcela Pasetti, University of Maryland, USA - Fidausi Qadri, ICDDRB, Bangladesh - Philippe Sansonetti, Institut Pasteur, France - Ann-Mari Svennerholm, Goteborg University, Sweden - Dani Cohen, Tel Aviv University, Israel - Shabir Mahdi, National Institute for Communicable Diseases, South Africa - Edward Ryan, Harvard University, USA - Samba Sow, University of Maryland, USA - Cecil Czerkinsky, International Vaccine Institute, Korea - Karen Kotloff, University of Maryland, USA - Kathy Neuzil, PATH, USA |
| WASH Interventions | Valerie Curtis  Director of the Hygiene Centre at the London School of Hygiene and Tropical Medicine | - Sharad Adhikary, WHO, India - Hamed Bakir, WHO-EMRO, Jordan - Sandy Cairncross, London School of Hygiene and Tropical Medicine, UK - Yolande Coombes, Water and Sanitation Program, Kenya - Barbara Evans, University of Leeds, UK - Steve Luby, ICDDRB, Bangladesh - Lyla Mehta, University of Sussex, UK - Mrs. Payden, WHO, India - Abdou-Salam Savadogo, WHO, Geneva - Yael Vellemen/Richard Carter, WaterAid, UK - Gijs Walraven, Aga Khan University, Pakistan - Kate Spooner, Water and Sanitation for Africa, UK - Mike Muller, University of Witwatersrand, South Africa |
| Case Management (Including ORS, Zinc, Antibiotics and Nutrition Therapy) | Mathuram Santosham  Director of the Health Systems Program, Director of the Center for American Indian Health, Professor of International Health and Pediatrics at the Johns Hopkins Bloomberg School of Public Health | - Alfred Bartlett, Save the Children, USA - Shinjini Bhatnagar, All India Institute of Medical Sciences, India - Alejandro Cravioto, ICDDRB, Bangladesh - Chris Duggan, Harvard University, USA - George Fuchs, University of Arkansas, USA - Adenike Grange, Otunba Tunwase National Paediatric Centre, Nigeria - Mathuram Santosham, Johns Hopkins University, USA - Evan Simpson, PATH, USA - Samba Sow, University of Maryland, USA - Renee Van der Weerdt, UNICEF, USA - Christa Fischer-Walker, Johns Hopkins University, USA - Olivier Fontaine, WHO, Switzerland - Sue Horton, University of Waterloo, Canada - Ronald Kleinman, Massachusetts General Hospital, USA |
| Emerging Interventions | Philip Sherman  Professor of Paediatrics, Microbiology, & Dentistry at the Hospital for Sick Children, recipient of Canada Research Chair (tier 1) in Gastrointestinal Disease  Alvin Zipursky  Chair and Scientific Director of Programme for Global Paediatric Research | - Stephen Freedman, SickKids Hospital, Canada - Mitchell Cohen, Cincinnati Children’s Medical Center, USA - Conrad Cole, Cincinnati Children’s Medical Center, USA - Alessio Fasano, University of Maryland, USA - Olivier Fontaine, WHO, Switzerland - Lori Holtz, Washington University in St. Louis, USA - Jose Armando Madrazo, Mexico - Marina Orsi, Hospital Italiano de Buenos Aires, Argentina - BS Ramakrishna, Christian Medical College, India - Hania Szajewska, Medical University of Warsaw, Poland - Alan Verkman, University of California San Francisco, USA - Anita Zaidi, Aga Khan University, Pakistan |
| Other Innovations | Evan Simpson  Program Officer for Enhanced Diarrhoeal Disease Control Initiative at PATH | - Elizabeth Blanton - Mark Guy, PATH, USA - David Marsh, Save the Children, USA - Ram Pavani, University of Buffalo, USA - Kate Schroder, Clinton Health Access Initiative, USA - Evan Simpson, PATH, USA - Anna Stratis, World Health Partners, India - Edward Ryan, Harvard University, USA - Shafiqul Sarker, ICDDRB, Bangladesh - Charles Larson, University of British Columbia, Canada |
| Monitoring and Evaluation | Mark Young  Senior Health Specialist, Policy and Evidence, United Nations Children's Fund (UNICEF) | - Malia Boggs, USAID, USA - Jai Das, Aga Khan University, Pakistan - Theresa Diaz, UNICEF, USA - Liliana Carvajal, UNICEF, USA - Shams El Arifeen, Bangladesh - Birger Forsberg, Karolinska Institute, USA - Dyness Kasungami, MCHIP, USA - Charles Larson, University of British Columbia, Canada - Vicki MacDonald, SHOPS-ABT Associates, USA - Modest Mulenga, Tropical Disease Research Centre, Zambia - Lynette Neufield, Micronutrient Initiative, Canada - Tim O’Brien, USAID, USA - Cynthia Boschi-Pinto, WHO, Switzerland - Mahesh Srinivas, Micronutrient Initiative, Canada - Nine Steensma, Clinton Health Access Initiative, USA - Mark Young, UNICEF, USA - Rana Hajjeh, Center for Disease Control, USA - Khassoum Diallo, UNICEF, Nairobi |

**Supplementary Table S17 – Description of Standard CHNRI Criteria**

| **Criteria** | **Sub-Questions:** |
| --- | --- |
| Answerability | 1. Is the research question well-framed with well-defined endpoints? 2. Based on the level of existing research capacity and the size of the research gap, would you say that a study can be designed to answer the research question? 3. Do you think that a study needed to answer the proposed research question would obtain ethical approval without major concerns? |
| Effectiveness | 1. Based on the best existing evidence and knowledge, would the intervention, which would be developed/improved through the proposed research be **efficacious**? 2. Based on the best existing evidence and knowledge, would the intervention, which would be developed/improved through the proposed research be **effective**? 3. If your answer to either Q1 or Q2 is positive, would you say that the evidence upon which these opinions are based is of high quality? |
| Deliverability | 1. Taking into account the level of difficultly with intervention delivery from the perspective of the intervention itself (i.e. design, standardization, safety); the infrastructure required (i.e. human resources, health facilities, communication and transport infrastructure); users of the intervention say that the endpoints of the research would be **deliverable** within the context of interest? 2. Taking into account the resources available to implement the intervention, would you say that the endpoints of the research would be **affordable** within the context of interest? 3. Would government capacity and partnership be essential to ensure the endpoints of the research would be **sustainable**? |
| Disease Burden Reduction | 1. Taking into account the results of conducted intervention trials, or for new interventions, the proportion of avertable burden under an “ideal scenario,” would you say that the successful reaching of research endpoints would have a capacity to remove 25% of the disease burden or more? 2. Taking into account the results of conducted intervention trials, or for new interventions, the proportion of avertable burden under an “ideal scenario,” would you say that the successful reaching of research endpoints would have a capacity to remove 50% of the disease burden or more? 3. Taking into account the results of conducted intervention trials, or for new interventions, the proportion of avertable burden under an “ideal scenario,” would you say that the successful reaching of research endpoints would have a capacity to remove 75% of the disease burden or more? |
| Effect on Equity | 1. Does the present distribution of the disease burden affect mainly the underprivileged in the population? 2. Would you say that mainly the underprivileged would be most likely to benefit from the results of the proposed research after its implementation? 3. Would you say that the proposed research has the overall potential to improve equity in disease burden distribution in the long-term (e.g. in 10 years)? |

**Supplementary Table S18 – Description of CHNRI Criteria for Monitoring and Evaluation Team**

| **Criteria** | **Sub-Questions** |
| --- | --- |
| Answerability | 1. Is the research question well-framed with well-defined endpoints? 2. Based on the level of existing research capacity and the size of the research gap, would you say that a study can be designed to answer the research question? 3. Do you think that a study needed to answer the proposed research question would obtain ethical approval without major concerns? |
| Importance/ Potential Impact | 1. Will the results of this research question fill an important knowledge gap? 2. Are the results from this research question likely to shape future planning and implementation? 3. Will the results from this research question be relevant to most countries? |
| Feasibility | 1. Is it likely that, in the context of interest, there will be sufficient capacity to carry out the results of this research? 2. Is it feasible to provide the training required for staff to carry out this research in the context of interest? 3. Is the cost and time required for this research reasonable within the context of interest? |
| Effect on Equity | 1. Will this research question cover measurements in the most impoverished populations? 2. Would you say that mainly the underprivileged would be most likely to benefit from the results of the proposed research question after its implementation? 3. Would you say that the proposed research has the overall potential to improve equity in disease burden reduction in the long-term (e.g. 10 years)? |

1. This question was longer in the original list, used shortened question provided to CHNRI workshop group [↑](#footnote-ref-1)
2. This question was longer in the original list, used shortened question provided to CHNRI workshop group [↑](#footnote-ref-2)
